# Diterpenoid Alkaloids from the Aerial Parts of *Aconitum flavum* Hand.-Mazz

**DOI:** 10.1007/s13659-021-00302-3

**Published:** 2021-04-16

**Authors:** Na Zhang, Fan Xia, Song-Yu Li, Yin Nian, Li-Xin Wei, Gang Xu

**Affiliations:** 1 State Key Laboratory of Phytochemistry and Plant Resources in West China and Yunnan Key Laboratory of Natural Medicinal Chemistry, Kunming Institute of Botany, Chinese Academy of Sciences, Kunming, 650201 China; 2 University of Chinese Academy of Sciences, Beijing, 100049 China; 3 Key Laboratory of Tibetan Medicine Research and Qinghai Provincial Key Laboratory of Tibetan Medicine Pharmacology and Safety Evaluation, Northwest Institute of Plateau Biology, Chinese Academy of Sciences, Xining, Qinghai China

**Keywords:** *Aconitum flavum* Hand.-Mazz, Diterpenoid alkaloids, Bioactivity

## Abstract

Sixteen diterpenoid alkaloids (DAs), including six aconitine-type alkaloids (**5** and **9** − **13**), seven 7,17-*seco*-aconitine-type alkaloids (**1** − **4**, **6** − **8**), two napelline-type alkaloids (**14** and **15**) as well as one veatchine-type alkaloid (**16**), were isolated from the aerial parts of *Aconitum flavum* Hand.-Mazz. In which, flavumolines A − D (**1** − **4**) were four new ones, and flavumoline E (**5**) was reported as natural compound for the first time. Their chemical structures were elucidated by the analysis of extensive spectroscopic data. The inhibitory activities of these isolates on Ca_v_3.1 low voltage-gated Ca^2+^ channel, NO production in LPS-activated RAW264.7cells, five human tumor cell lines, as well as acetylcholinesterase (AChE) were tested.

**Supplementary Information:**

The online version contains supplementary material available at 10.1007/s13659-021-00302-3.

## Introduction

*Aconitum* species represent a large genus in the Ranunclaceae family [1]. It is estimated that there are more than 350 species of *Aconitum* all over the world, which are widely distributed in the northern temperate zone, of which 173 species are endemic to mainland China [1]. Previously chemical and pharmacological studies shown that the diterpenoid alkaloids (DAs) were the main pharmacological constituents of this genus [2]. So far, around 1300 natural DAs, categorized into C_20_-, C_19_-, and C_18_- families depending on the number of contiguous carbon atoms, have been reported [3], [4], [5]. Meanwhile, they have been the targets of medicinal chemists for a broad range of confirmed pharmacological properties including analgesic, antiarrhythmic, anti-inflammatory, hypotensive, neuroprotective and so on [6].

*Aconitum flavum* Hand.-Mazz, known as a perennial herb, is mainly distributed in Qinghai, Gansu, and other northwest places in China [7]. As one of the most vital Chinese traditional ethnic minority folk medicine, the roots were commonly used for the treatment of traumatic injuries, rheumatic arthritis, and some other inflammations [7]. Previously phytochemical investigations of this plant have resulted in the isolation of more than 20 DAs from the roots, such as aconitine, mesaconitine, and deoxyaconitine, which were not only considered as the predominant toxic components of this folk medicine, but simultaneously, the significant active constituents [8]. In this study, sixteen DAs including four new 7,17-*seco*-aconitine-type DAs (flavumolines A − D, **1** − **4**), one new natural compound (**5**), together with 11 known analogues (**6** − **16**) were identified. These compounds could be divided into four different types: six aconitines (**5** and **9** − **13**), seven 7,17-*seco*-aconitines (**1** − **4** and **6** − **8**), two napellines (**14** and **15**) as well as one veatchine-type alkaloid (**16**). All of these isolated compounds were evaluated for their inhibitory effects on Ca_v_3.1 low voltage-gated Ca^2+^ channel, NO production in LPS-activated RAW264.7 cells, five human tumor cell lines, and acetylcholinesterase (AChE). Reported herein, the isolation, structural determination, and biological activity of these compounds were thoroughly described.

## Results and Discussion

The MeOH extract was subjected to repeated column chromatography to yield four new ones (flavumolines A − D, **1**–**4**), one new natural compound (**5**), together with 11 known analogues 13-hydroxylfranchetine (**6**) [9], vilmorisine (**7**) [10], franchetine (**8**) [11], 3-acetylaconitine (**9**) [12], 15-dehydroxyldecludine A (**10**) [13], pubescensine (**11**) [14], 3-O-acetyl-20-deethyl-20-formylaconitine (**12**) [15], 14*α*-benzoyloxy-*N*-ethyl-3*α*,13*β*,15*α*-trihydroxy-1*α*,6*α*,8*β*,16*β*,18-pentamethoxyaconitane (**13**) [16], 15-acetylsongoramine (**14**) [17], dehydrolucidusculine (**15**) [18], and veatchine azomethine (**16**) [19].

Flavumoline A (**1**) was obtained as a white amorphous powder with the molecule formula as C_29_H_35_NO_8_ determined on the basis of HRESIMS at *m/z* 548.2259 [M + Na]^+^ (calcd for C_29_H_35_NO_8_Na, 548.2255). The presence of hydroxyl (3431 cm^−1^) and carbonyl (1672 cm^−1^) units was deduced from the IR spectrum. The ^13^C NMR and DEPT spectra displayed 29 carbons signals, which were divided into five methylenes, nine methines, and five quaternary carbons as well as the signals for a benzoyl and three methoxy groups. Detailly, the characterized signals for a franchetine-type C_19_-DA core could be distinguished as follows: one representative 6,17-*epoxy* unit (consisted of two oxygenated methines at *δ*_C_ 83.1, C-17 and *δ*_C_ 76.2, C-6), two quaternary carbons at *δ*_C_ 48.9 (C-4) and 51.0 (C-11), and three methines at *δ*_C_ 46.5 (C-5), 44.3 (C-9), and 47.1 (C-10), together with the typical C-7/C-8 trisubstituted double bond (*δ*_C_ 128.0, d, C-7 and *δ*_C_ 137.4, s, C-8) [3]. The ^1^H NMR spectrum of **1** also verified resonances assignable to two oxygenated methines at *δ*_H_ 4.62 (d, *J* = 4.6 Hz, H-17) and *δ*_H_ 4.71 (d, *J* = 6.1 Hz, H-6), and a singlet at *δ*_H_ 5.78 (d, *J* = 5.8 Hz, H-7). These evidences, conjugated with the fact that a number of DAs have been reported as the major constituents of this genus, suggested that compound **1** was assigned to be a typical franchetine-type C_19_-DA [20]. This deduction could be further confirmed by the HMBC correlated signals of H-5 with C-4/C-7/C-10/C-11/C-17/C-18/C-19, H-9 with C-12/C-13/C-14, and H-10 with C-1/C-5/C-9/C-11/C-12/C-17. Furthermore, the 6,17-*epoxy* unit was confirmed by the correlations from H-17 to C-6/C-11 and H-6 to C-5/C-7/C-8/C-11/C-17. In addition, the correlations of H-3, H-5, H-17, and H-18 with C-19, combined with correlations from N–H (*δ*_H_ 6.29, d, *J* = 4.7 Hz) to C-4/C-11/C-19/C-17 confirmed the amide moiety between nitrogen atom and C-19 (Fig. 2).

Moreover, the benzoyl group was placed at C-14 according to the HMBC correlation from H-14 (*δ*_H_ 5.07, s) to carbonyl (*δ*_C_ 166.7, s). Three methoxy units were located at C-1, C-16, and C-18 on the basis of the HMBC correlated signals from OCH_3_-1 (*δ*_H_ 3.41, s) to C-1 (*δ*_C_ 85.2, d), from OCH_3_-16 (*δ*_H_ 3.50, s) to C-16 (*δ*_C_ 85.8, d), and from OCH_3_-18 (*δ*_H_ 3.31, s) to C-18 (*δ*_C_ 74.1, t), respectively. Besides, a hydroxyl group should be located at C-13 corresponding to the HMBC correlations from OH to C-12/C-13/C-16.

Biogenetically, the configurations of H-5, 9, 10, could be ascribed to *β-*orientation whereas H-17 to *α*-orientation, which could be confirmed by ROESY correlations of H-5/9/10/12*β* and H-17/12*α*, respectively [21]. Then, the *α*-orientation of the benzoyl group at C-14 was determined on the ROESY correlations between H-14 and H-9. And the *α-*orientation of the OCH_3_-1 was identificated by the ROESY correlations between H-1/H-10. In addition, the correlations of H-17/H-12*α*/H-16 and 13-OH/H-14 demonstrated the *β*-orientation of OCH_3_-16 and 13-OH (Fig. 3, the dashed lines and solid lines to represent *α*-orientation and *β*-orientation, respectively). Therefore, the structure of **1** was determined and named flavumoline A.

**Fig. 1:**
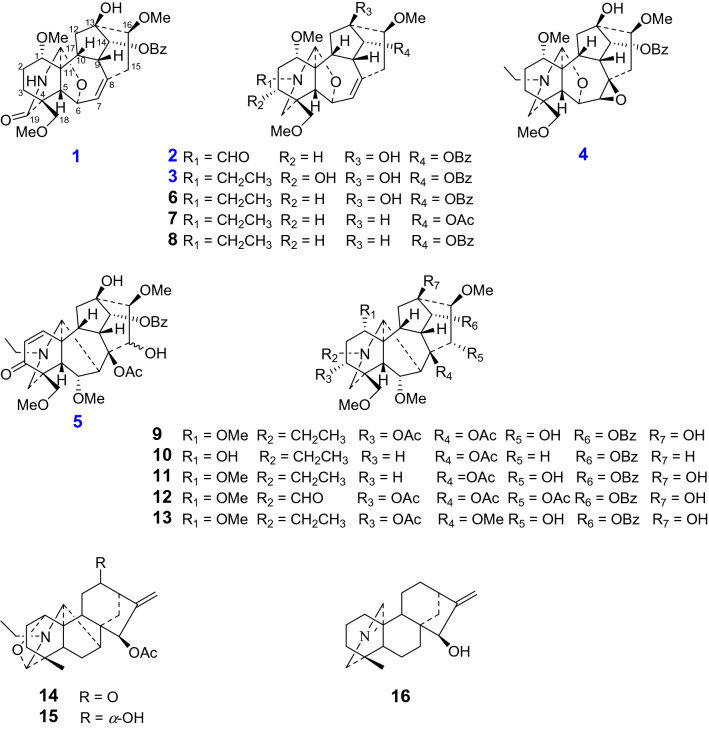
Structures of compounds **1**–**16** from *A. flavum* Hand.-Mazz

**Fig. 2:**
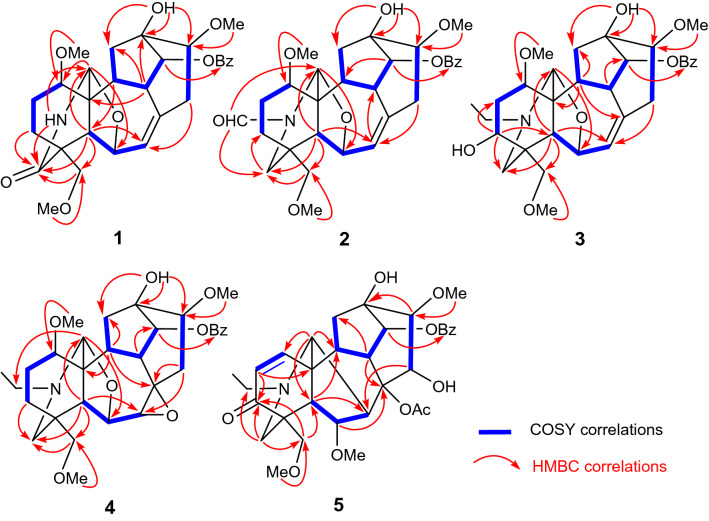
Key ^1^H–^1^H COSY and HMBC correlations of compounds **1** − **5**

The molecular formula of compounds **2** (C_30_H_37_NO_8_) and **3** (C_31_H_41_NO_8_) were established on the analysis of HRESIMS and NMR data. The NMR data of **2** showed the presence of six methylenes, eight methines, three quaternary carbons, and a characteristic trisubstituted intra-annular double bond, in addition to a benzoyl, an aldehyde group and three methoxy groups. All of these spectroscopic data suggested that **2** was an analogue of 13-hydroxylfranchetine (**6**) [9]. The main difference was the presence of a *N*-CHO moiety in compound **2** instead of *N*-ethyl group in **6**, which was verified by 1D NMR data and HMBC correlations. The HMBC correlations (Fig. 2) from the singlet for aldehyde group (*δ*_H_ 7.95, s) to C-19 and C-17 suggested that the aldehyde group was placed at *N*-atom. On the analysis of ROESY data, the correlations of H-14/H-9, H-16/H-12*α*/H-17, H-1/H-10 confirmed the *α*-orientation of benzoyl group at C-14, *β*-orientation of methoxy at C-16, and *α*-orientation of methoxy at C-1, respectively. Compared with **2**, compound **3** possessed a typical *N*-ethyl moiety, which were elucidated by the HMBC correlations from H-21 to C-17, C-19. A hydroxyl located at C-3 (*δ*_C_ 71.0, d) confirmed by the ^1^H-^1^H COSY correlation of H-2/H-3 and HMBC correlations of H-3 with C-2/5/19. Additionally, the ROESY correlations of H-3/H-1/H-5 confirmed *α*-orientation of 3-OH. Therefore, the structures of flavumoline B (**2**) and flavumoline C (**3**) were confirmed as shown above (Fig. 1).

Flavumoline D (**4**) was a colorless oily liquid with molecule formula of C_31_H_41_NO_8_ as deduced by analysis of the ion peak at *m/z* 556.6689 [M + H]^+^ in the HRESIMS (calcd for C_31_H_42_NO_8_, 556.6702), which indicated 10 degrees of unsaturation. The ^13^C NMR and DEPT data showed six methylenes, nine methines, four quaternary carbons, as well as the signals for a benzoyl group, three methoxy groups, and a typical *N*-ethyl group. Side by side, comparison of its NMR data with those of **6**, a known franchetine-type DA isolated previously, indicated that the structure of **4** was similar with that of **6** [9], except that the C-7/C-8 double bond was replaced by 7,8-*epoxy* unit (*δ*_C_ 64.0, C-7 and *δ*_C_ 59.0, C-8). The HMBC correlations of H-5/H-15 with C-7 and H-7/H-9/H-10/H-15 with C-8 confirmed the 7,8-*epoxy* unit. In addition, the remained three methoxy groups, one hydroxyl, and one benzoyl were elucidated to be attached on C-1, C-16, C-18, C-13, and C-14, respectively, as deduced by the HMBC correlations of OCH_3_-1 (*δ*_H_ 3.35, s) with C-1 (*δ*_C_ 86.0, d), OCH_3_-16 (*δ*_H_ 3.42, s) with C-16 (*δ*_C_ 82.6, d), OCH_3_-18 (*δ*_H_ 3.30, s) with C-18 (*δ*_C_ 79.0, t), hydroxyl group (*δ*_H_ 3.92, s) with C-12/C-13/C-16, and H-14 (*δ*_H_ 5.11, d, *J* = 3.0 Hz) with the carbonyl (*δ*_C_ 166.7, s) of the benzoyl group (Fig. 2).

**Fig. 3:**
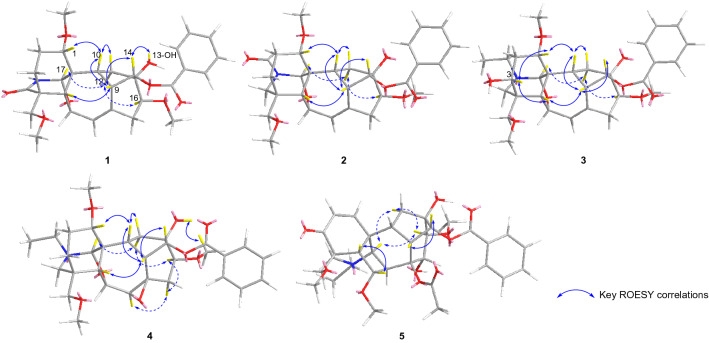
Key ROESY correlations of compounds **1** − **5**

The relative configuration of **4** was confirmed by the analysis of ROESY spectrum. At first, the correlation from H-14 to H-9 implied the benzoyl group at C-14 was *α*-oriented. The configurations of methoxyl groups were confirmed as 1*α*-OCH_3_ and 16*β*-OCH_3_ on account of the correlations of H-1/H-10 and H-16/H-12*α*/H-17. Then, the remained hydroxyl group at C-13 was established as *β*-oriented according to its correlation with 16-OCH_3_. On the basis of correlations of H-7/H-15*α*/H-16, the configuration of 7,8-*epoxy* unit was identified as *β*-orientation (Fig. 3). Hence, the structure of **4** was established as above.

Flavumoline E (**5**), was isolated as white amorphous powder. Its molecular formula was established as C_33_H_41_NO_10_ on the basis of HRESIMS at *m/z* 612.2799 [M + H]^+^ (calcd for C_33_H_42_NO_10_, 612.2803), in combination with NMR spectroscopic data. The IR spectrum of **5** showed absorptions due to the hydroxyl (3487 cm^−1^) and carbonyl (1676 cm^−1^) functionalities. The ^13^C NMR and DEPT spectra of **5** displayed 33 carbon resonances including five quaternary carbons (one carbonyl at *δ*_C_ 200.5, s, C-3, two oxygenated carbons at *δ*_C_ 92.1, C-8 and *δ*_C_ 74.3, C-13), eleven methines, three methylenes, and 14 other signals attributable to a benzoyl, a typical *N*-ethyl group, an acetoxyl, and three methoxy groups. By carefully analyzing the characteristic resonances of a *N*-ethyl group at *δ*_C_ 49.2, t (C-21) and *δ*_C_ 13.2, q (C-22), a nitrogen-bearing methine and methylene at *δ*_C_ 60.8, d (C-17) and *δ*_C_ 51.0, t (C-19), an *α,β*-unsaturated ketone moiety at *δ*_H_ 6.27, 6.46 (each 1H, d, *J* = 10.3 Hz) together with the C-atom signals at *δ*_C_ 132.3, 147.3, and 200.5 were clearly observed. The ^1^H NMR spectrum also verified resonances assignable to a nitrogen-bearing methine and methylene at *δ*_H_ 2.76 (s, H-17), *δ*_H_ 2.62 (d, *J* = 11.4 Hz, H-19a) and 2.38 (overlap, H-19b), as well as a representative *N*-ethyl group at *δ*_H_ 2.39, 2.69 (m, H-21) and *δ*_H_ 1.00 (t, *J* = 7.1 Hz, H-22). These evidences suggested that **5** should be a typical aconitine-type C_19_-DA [22]. This deduction could be further confirmed by the HMBC correlations of H-17 with C-5/C-6/C-8/C-10/C-11/C-19, H-19 with C-3/C-4/C-5/C-17/C-18/C-21, H-5 with C-4/C-7/C-10/C-11/C-17/C-18/C-19, H-9 with C-8/C-12/C-13/C-14/C-15, H-10 with C-8/C-9/C-12/C-13/C-17, toghther with the connection of C-17, C-19, and C-21 to the same nitrogen atom evidenced by the HMBC correlations from H-19 to C-17 and C-21, from H-21 to C-17 and C-19 (Fig. 2).

According to the HMBC spectrum, the correlations between one OH group (*δ*_H_ 4.01, s) and C-12/C-13/C-16, another OH group (*δ*_H_ 4.47, d, *J* = 3.0 Hz) and C-13/C-15/C-16, OAc (*δ*_H_ 1.45, s) and C-8, H-14 (*δ*_H_ 4.98, d, *J* = 5.0 Hz) and carbonyl of benzoyl group, identified the location of these substituent groups at C-13, C-15, C-8, and C-14, respectively. Additionally, the substitutions of 6-OCH_3_, 16-OCH_3_, and 18-OCH_3_ were identified by the correlations from OCH_3_-6 to C-6, OCH_3_-16 to C-16, OCH_3_-18 to C-18. The HMBC correlated resonances between H-1 and C-10/C-17, H-2 and C-4/C-11, H-18a,b/H-19a,b and C-3, demonstrated that the C = C bond was located between C-1 and C-2 and the carbonyl group at C-3 (200.5, s).

Biosynthetically, the configurations of H-5, 9, 10, 8-OAc, 13-OH could be ascribed to *β-*orientation and H-17 to *α*-orientation, respectively, due to the caged core of aconitine-type C_19_-DA [23]. Then, the configurations of two methoxy groups and one benzoyl at C-6, 16, and 14 were confirmed as *α*, *β*, and *α*, respectively, according to the correlations between H-6/H-5, H-16/H-12*α*/H-17 and H-14/H-9 (Fig. 3). However, the configuration of 15-OH has not been confirmed yet because of the simultaneous correlations of H-15 with H-7/H-16/16-OCH_3_ and 15-OH with H-7/H-16/16-OCH_3._ Eventually, the structure of **5** was identified as shown in Fig. 1, which was reported as a natural compound for the first time according to the literature on synthesis [24].

Since diterpenoid alkaloids in *Aconitum* were reported to commonly treat traumatic injury, arrhythmia, and rheumatism, in which ion channels or inflammation were involved in the pathophysiological process and inhibitors of ion channels or NO release were considered as potential agents for the treatment of these diseases [25], these isolated compounds were evaluated for their inhibitory effects on T-type ion channels using the whole-cell recording patch clamp method, NO production in LPS-activated RAW264.7 cells using Griess assay [26], on five human tumor cell lines [27], as well as acetylcholinesterase (AChE) [28]. As a result, compound **8** (30 μM) exhibited 64.5% inhibitory rate on Ca_v_3.1 low voltage-gated Ca^2+^ channel. Compounds **3**, **4**, **5**, **6**, **7**, and **11** showed potential inhibitory effects on NO production ranging from 20% to 32% at 50 μM. Additionally, compound **5** showed potential inhibitory effects on four human tumor cell lines HL-60, A-549, SMMC-7721, MCF-7 with IC_50_ value as 16.88, 33.11, 23.97, 24.21 μM in vitro, and no compound showed inhibitory effect on AChE.

## Experimental

### General Experimental Procedures

Optical rotations were measured on a Jasco P-1020 polarimeter. UV spectra were detected on a Shmadzu UV-2401PC spectrometer. IR spectra were determined on a Bruker FT-IR Tensor-27 infrared spectrophotometer with KBr disks. All 1D and 2D NMR spectra were recorded on Bruker DRX-600 spectrometers using TMS as an internal standard. Unless otherwise specified, chemical shifts (*δ*) were expressed in ppm with reference to the solvent signals. ESIMS and HRESIMS analysis were carried out on Waters Xevo TQS and Aglient G6230 TOF mass spectrometers, respectively. Semi-preparative HPLC was performed on a Waters 2695 HPLC with a 5C_18_-MS-II column [4.6 × 150 mm]. Silica gel (100–200, 200–300 mesh, Qingdao Marine Chemical Co., Ltd., People’s Republic of China), and MCI gel (75–150 μm, Mitsubishi Chemical Corporation, Tokyo, Japan) were used for column chromatography. Fractions were monitored by TLC (GF 254, Qingdao Marine Chemical Co., Ltd.), and spots were visualized under a UV lamp at 254 nm or by spraying the Dragendorff’ reagent and heating silica gel plates sprayed with 10% H_2_SO_4_ in EtOH.

### Plant Material

The plants of *A. flavum* were collected in June 2019 from the Sanjiangyuan Nature Reserve of Guoluo, Qinghai Province, People’s Republic of China. Plant identity was verified by prof. Li-Xin Wei. A voucher specimen (No. 201901H19) was deposited in the Kunming Institute of Botany.

### Extraction and Isolation

Air-dried and powdered plant material (aerial parts) of *A. flavum* (45 kg) was extracted three times (3 × 150 L) with MeOH at room temperature and then concentrated to 3.88 kg under reduce pressure. The crude extract was suspended in 1% HCl followed by basification with 10% aqueous NH_4_OH (pH 9** − **10) and subsequently, extracted with ethyl acetate to afford crude alkaloids (808 g). The total alkaloids fraction was separated on a silica gel column (CHCl_3_/CH_3_OH, 100:1 − 1:1) to yield eight fractions (Fr. 1 − 8). Then Fr. 1 (35 g) was separated on a silica gel column (PE/EtOAc/DEA, 50:1:1 − 3:1:1) to yield six sub-fractions (Fr. 1a − 1f). Fr. 1a (2.3 g) was separated on a silica gel column (PE/EtOAc, 80:1–3:1) and then semi-preparative HPLC (Waters 2695 C18, i.d. 150 × 10 mm, 5 μm, 3.0 ml/min, UV 210 nm) using MeOH/H_2_O (85/15, v/v) as the mobile phase afforded **6** (57 mg), **7** (10 mg) and **8** (23 mg). Fr. 1b (2.7 g) was separated on a silica gel column (PE/EtOAc, 60:1–3:1) followed by preparative HPLC using MeOH/H_2_O (75/25, v/v) to yield **4** (128 mg), **14** (15 mg), **15** (9.7 mg) and **16** (6 mg). Fr. 1c (3 g) was separated on a silica gel column (PE/EtOAc, 50:1–3:1), followed by semi-preparative HPLC using MeOH/H_2_O (70/30, v/v) as the mobile phase to afford **10** (8 mg), **11** (3 mg) and then yield **9** (230 mg) by recrystallizaiton. Fr. 1d (7 g) was separated on a silica gel column (PE/EtOAc, 50:1–1:1) yielded **12** (50 mg) and then on semi-preparative HPLC using MeOH/H_2_O (70/30, v/v) as the mobile phase to afford **13** (10 mg). Fr. 1e (5.5 g) yielded **2** (7.3 mg) and **3** (20.1 mg) by preparative HPLC using MeOH/H_2_O (70/25, v/v) as mobile phase, subsequently, afforded **5** (4.7 mg) and **1** (2 mg) isolated by Sephadex II column using acetone.

#### Flavumoline A (**1**)

White powder; [*α*]_D_^19^ −4.9 (*c* 0.175, MeOH); IR (KBr) *ν*_max_: 3431, 2975, 2933, 2828, 1718, 1672, 1282, 1099 cm^−1^; For ^13^C NMR data (see Table 1) and ^1^H NMR data (see Table 2). ESIMS *m/z* 526 [M + H]^+^; HRESIMS *m/z*: 548.2259 [M + Na]^+^ (calcd for C_29_H_35_NO_8_Na, 548.2255).

**Table 1:** ^13^C NMR (150 MHz) spectroscopic data for compounds **1** − **5** (in CDCl_3_)

No	**1**	**2**	**3**	**4**	**5**
1	85.2	85.1	83.9	86.0	147.3
2	25.1	23.6	33.0	24.2	132.3
3	31.0	32.3	71.0	32.4	200.5
4	48.9	36.5	42.0	36.9	49.3
5	46.5	46.8	45.2	43.0	48.6
6	76.2	76.5	74.6	76.0	82.5
7	128.0	127.9	128.9	64.0	45.1
8	137.4	137.4	136.0	59.0	92.1
9	44.3	44.0	43.9	44.3	43.3
10	47.1	47.6	46.6	44.4	37.6
11	51.0	51.3	50.3	49.1	51.1
12	38.6	38.8	38.8	38.5	38.2
13	77.3	77.2	77.3	76.8	74.3
14	83.0	83.0	83.4	82.8	79.1
15	39.1	39.0	39.0	36.9	78.9
16	85.8	85.7	85.9	82.6	90.2
17	83.1	86.9	92.1	94.4	60.8
18	74.1	77.6	75.8	79.0	72.0
19	174.9	41.9	45.5	52.2	51.0
21	–	160.7	49.0	49.0	49.2
22	–	–	13.0	13.0	13.2
1-OCH_3_	57.3	57.0	57.1	57.1	–
6-OCH_3_	–	–	–	–	58.5
16-OCH_3_	58.2	58.1	58.1	58.1	61.6
18-OCH_3_	59.5	59.5	59.5	59.5	59.3
-OBz	166.7	166.7	166.7	166.7	166.1
1’	130.1	130.0	130.2	130.0	129.7
2′,6’	129.8	129.8	129.8	129.8	129.8
3′,5’	128.5	128.5	128.5	128.4	129.0
4’	133.2	133.2	133.1	133.2	133.7
8-OAc	–	–	–	–	172.6
	–	–	–	–	21.6

**Table 2:** ^1^H NMR (600 MHz) spectroscopic data for compounds **1** − **5** (in CDCl_3_)

No	**1** (*J* in Hz)	**2** (*J* in Hz)	**3** (*J* in Hz)	**4** (*J* in Hz)	**5** (*J* in Hz)
1	3.58 m	3.52 m	3.38 m	3.21 m	6.46 d (10.3)
2	2.20 m 1.74 m	2.05 m 1.52 m	2.52 m 2.22 m	2.38 m 1.93 m	6.27 d (10.3)
3	1.83 m 1.78 m	1.79 m 1.60 m	3.85 dd (4.9 12.3)	1.80 m 1.44 m	–
4	–	–	–	–	–
5	2.68 m	2.47 s	2.19 s	1.82 s	3.11 d (6.4)
6	4.71 d (6.1)	4.69 d (6.0)	4.39 d (6.1)	4.58 d (4.5)	4.11 d (6.4)
7	5.78 d (5.8)	5.77 d (5.76)	5.76 d (5.8)	2.90 d (4.2)	3.04 br s
8	–	–	–	–	–
9	3.28 br s	3.25 br s	3.14 br s	2.70 m	2.98 m
10	2.73 m	2.71 m	2.60 m	2.56 m	2.37 m
11	–	–	–	–	–
12	2.14 m 2.07 m	2.20 m 2.00 m	2.08 m 1.99 m	2.58 overlap 2.21 m	2.44 m
					1.74 m
13	–	–	–	–	–
14	5.07 s	5.06 br s	5.01 br s	5.11 d (3.0)	4.98 d (5.0)
15	2.99 m 2.70 m	2.98 m 2.69 overlap	2.92 m 2.62 m	2.78 m 1.68 m	4.54 dd (2.7 5.3)
16	3.35 m	3.37 m	3.31 m	3.46 m	3.31 d (5.4)
17	4.62 d (4.6)	4.96 s	4.35 s	4.49 br s	2.76 s
18	3.68 d (9.7) 3.60 d (9.7)	3.22 d (9.4) 3.10 d (9.4)	3.32 overlap 3.17 d (9.4)	3.09 br s	3.91 d (8.5)
					3.65 d (8.5)
19	–	3.74 d (13.7) 2.66 overlap	2.87 d (11.3)	2.52 overlap 2.11 d (11.3)	2.62 d (11.4)
	–		1.73 d (11.3)		2.38 overlap
21	–	7.95 s	2.57 m 2.38 m	2.65 m 2.48 m	2.69 m
					2.39 overlap
22	–	–	1.01 t (7.3)	1.05 t (7.3)	1.00 t (7.1)
1-OCH_3_	3.41 s	3.33 s	3.34 s	3.35 s	–
6-OCH_3_	–	–	–	–	3.22 s
16-OCH_3_	3.50 s	3.51 s	3.44 s	3.42 s	3.77 s
18-OCH_3_	3.31 s	3.28 s	3.28 s	3.30 s	3.27 s
2′,6’	8.10 d (7.6)	8.08 d (7.8)	8.05 d (7.8)	8.08 d (7.6)	8.04 d (7.4)
3′,5’	7.47 t (7.6)	7.47 t (7.8)	7.43 t (7.8)	7.48 t (7.6)	7.48 t (7.4)
4’	7.59 t (7.6)	7.59 t (7.8)	7.55 t (7.8)	7.58 t (7.6)	7.60 t (7.4)
15-OH	–	–	–	–	4.47 d (3.0)
13-OH	3.95 s	3.93 s	3.89 s	3.92 s	4.01 s
N–H	6.29 d (4.7)	–	–	–	–
CHO	–	7.95 s	–	–	–
8-OAc	–	–	–	–	1.45 s

#### Flavumoline B (**2**)

White powder; [*α*]_D_^18^ −12.5 (*c* 0.100, MeOH); IR (KBr) *ν*_max_: 3447, 2938, 2882, 2828, 1717, 1670, 1280, 1099 cm^−1^; For ^13^C NMR data (see Table 1) and ^1^H NMR data (see Table 2). ESIMS *m/z* 540 [M + H]^+^; HRESIMS *m/z*: 562.2408 [M + Na]^+^ (calcd for C_30_H_37_NO_8_Na, 562.2411).

#### Flavumoline C (**3**)

White powder; [*α*]_D_^18^ −9.7 (*c* 0.103, MeOH); IR (KBr) *ν*_max_: 3534, 3402, 2974, 2824, 1717, 1280, 1103 cm^−1^; For ^13^C NMR data (see Table 1) and ^1^H NMR data (see Table 2). ESIMS *m/z* 556 [M + H]^+^; HRESIMS *m/z*: 556.2905 [M + H]^+^ (calcd for C_31_H_42_NO_8_Na, 556.2905).

#### Flavumoline D (**4**)

Colorless oily liquid; [*α*]_D_^19^_19_ −3.9 (*c* 0.101, MeOH); IR (KBr) *ν*_max_: 3437, 2973, 2932, 2826, 1719, 1279, 1101 cm^−1^; For ^13^C NMR data (see Table 1) and ^1^H NMR data (see Table 2). ESIMS *m/z* 556 [M + H]^+^; HRESIMS *m/z*: 556.6689 [M + H]^+^ (calcd for C_31_H_42_NO_8_, 556.6702).

#### Flavumoline E (**5**)

White powder; [*α*]_D_^18^ + 4.4 (*c* 0.122, MeOH); IR (KBr) *ν*_max_: 3487, 2973, 2936, 1721, 1676, 1280 cm^−1^; For ^13^C NMR data (see Table 1) and ^1^H NMR data (see Table 2). ESIMS *m/z* 612 [M + H]^+^; HRESIMS *m/z* 612.2799 [M + H]^+^ (calcd for C_33_H_42_NO_10_, 612.2803).

## Bioassays

### T-Type Ion Channel Inhibitory Activity Assay

All experiments were performed at room temperature (~ 22 °C). Pipettes were fabricated from borosilicate glass (World Precision Instru-ments) using a micropipette puller (P-1000, Sutter Instrument), and were fire-polished to resistances of 2 ~ 4 M for whole-cell recording. Whole-cell currents were elicited by 150 ms depolarization to − 40 mV at 4 s intervals from a holding potential (HP) of − 100 mV. Currents were amplified by Axopatch 200B and digitized by Digidata 1440A (Molecular Devices). Currents were low-pass filtered at 2 kHz and sampled at 10 kHz. pCLAMP 10 software (Molecular Devices) was used for data acquisition and analysis. The extracellular solutions contained (in mM) 142 CsCl, 1 MgCl_2_, 2 CaCl_2_, 10 Glucose and 10 HEPES (pH 7.4 adjusted with CsOH). The intracellular solutions contained (in mM) 127 Cs-methanesulphonate, 2MgCl_2_, 2Na_2_ATP, 10 HEPES and 11 EGTA (pH 7.4 adjusted with CsOH). The tested compounds (30 μM) were added.

### Anti-Inflammatory Activity Assay

The murine macrophage cell line RAW264.7 was obtained from Cell Bank of Chinese Academy of Sciences. RAW264.7 cells were seeded in 96-well cell culture plates (1.5 × 10^5^ cells/well) and treated with serial dilutions of the compounds with a maximum concentration of 50 μM in triplicate, followed by stimulation with 1 μg/mL LPS (Sigma) for 18 h. NO production in the supernatant was assessed by Griess reagents (Sigma). The absorbance at 570 nm was measured with a microplate reader (Thermo, Waltham, MA, USA). N^G^-Methyl-L-arginine acetate salt (L-NMMA, Sigma) was used as a positive control [29]. The viability of RAW264.7 cells was evaluated by the MTS assay simultaneously to exclude the interference of the cytotoxicity of the test compounds.

### Cytotoxicity Assay

The human tumor cell lines HL-60, SMMC-7721, A-549, MCF-7, and SW-480 were obtained from ATCC (Manassas, VA, USA). Cells were cultured in RMPI-1640 or DMEM medium (Biological Industries, Kibbutz Beit-Haemek, Israel) supplemented with 10% fetal bovine serum (Biological Industries) at 37 °C in a humidified atmosphere with 5% CO_2_. The cytotoxicity assay was evaluated by the 3-(4,5-dimethylthiazol-2-yl)-5-(3-carboxymethoxyphenyl)-2-(4-sulfophenyl)-2H-tetrazolium, inner salt (MTS) (Promega, Madison, WI, USA) assay [30]. Briefly, cells were seeded into each well of a 96-well cell culture plate. After 12 h of incubation at 37 °C, the test compound (40 μM) was added. After incubated for 48 h, cells were subjected to the MTS assay. Compounds with a growth inhibition rate of 50% were further evaluated at concentrations of 0.064, 0.32, 1.6, 8, and 40 μM in triplicate, with cisplatin and paclitaxel (Sigma, St. Louis, MO, USA) as positive controls. The IC_50_ value of each compound was calculated with Reed and Muench’s method [31].

### AChE Inhibitory Activity Assay

Acetylcholinesterase (AChE) inhibitory activity of the compounds isolated was assayed by the spectrophotometric method developed by Ellman et al.[32] with slightly modification. *S*-Acetylthiocholine iodide, *S*-butyrylthiocholine iodide, 5,5′-dithio-bis-(2-nitrobenzoic) acid (DTNB, Ellmans’ reagent), acetylcholinesterase derived from human erythrocytes were purchased from Sigma Chemical. Compounds were dissolved in DMSO. The reaction mixture (totally 200 *μ*L) containing phosphate buffer (pH 8.0), test compound (50 μM), and acetyl cholinesterase (0.02U/mL), was incubated for 20 min (37 °C). Then, the reaction was initiated by the addition of 40 μL of solution containing DTNB (0.625 mM) and acetylthiocholine iodide (0.625 mM) for AChE inhibitory activity assay, respectively. The hydrolysis of acetylthiocholine was monitored at 405 nm every 30 s for one hour. Tacrine was used as positive control with final concentration of 0.333 μM. All the reactions were performed in triplicate. The percentage inhibition was calculated as follows: (%) inhibition = (E—S)/E × 100 (E is the activity of the enzyme without test compound and S is the activity of enzyme with test compound).

## Supplementary Information

Below is the link to the electronic supplementary material.

Supplementary file1 (doc 9284 kb)
