# Supplementary material for: Diterpenoid Alkaloids from the Aerial Parts of Aconitum flavum Hand.-Mazz
Source: Nat Prod Bioprospect. 2021 Apr 16;11(4):421–9. doi: 10.1007/s13659-021-00302-3 (PMC8275754; doi:10.1007/s13659-021-00302-3)
Supplement: Supplementary file 1 — Supplementary file1 (doc 9284 kb) [file 13659_2021_302_MOESM1_ESM.doc]

**Diterpenoid alkaloids from the aerial parts of** ***Aconitum flavum* Hand.-Mazz**

Na Zhang^1,2^, Fan Xia^1^, Song-Yu Li^1,2^, Yin Nian^1^, Li-Xin Wei^3^, Gang Xu^1*^

*^1^ State Key Laboratory of Phytochemistry and Plant Resources in West China and Yunnan Key Laboratory of Natural Medicinal Chemistry, Kunming Institute of Botany, Chinese Academy of Sciences, Kunming 650201, China*

*^2^University of Chinese Academy of Sciences, Beijing 100049, China*

*^3^Key Laboratory of Tibetan Medicine Research and Qinghai Provincial Key Laboratory of Tibetan Medicine Pharmacology and Safety Evaluation, Northwest Institute of Plateau Biology, Chinese Academy of Sciences, Xining, Qinghai, China*

** Corresponding author.*

*E-mail address:* ^Gang Xu^: [xugang008@mail.kib.ac.cn](mailto:xugang008@mail.kib.ac.cn)

Supplementary data

**Figure S1–S8.**The original NMR and MS spectra of compound **1**

**Figure S9–S16.**The original NMR and MS spectra of compound **2**

**Figure S17–S24.**The original NMR and MS spectra of compound **3**

**Figure S25–S32.**The original NMR and MS spectra of compound **4**

**Figure S33–S40.**The original NMR and MS spectra of compound **5**


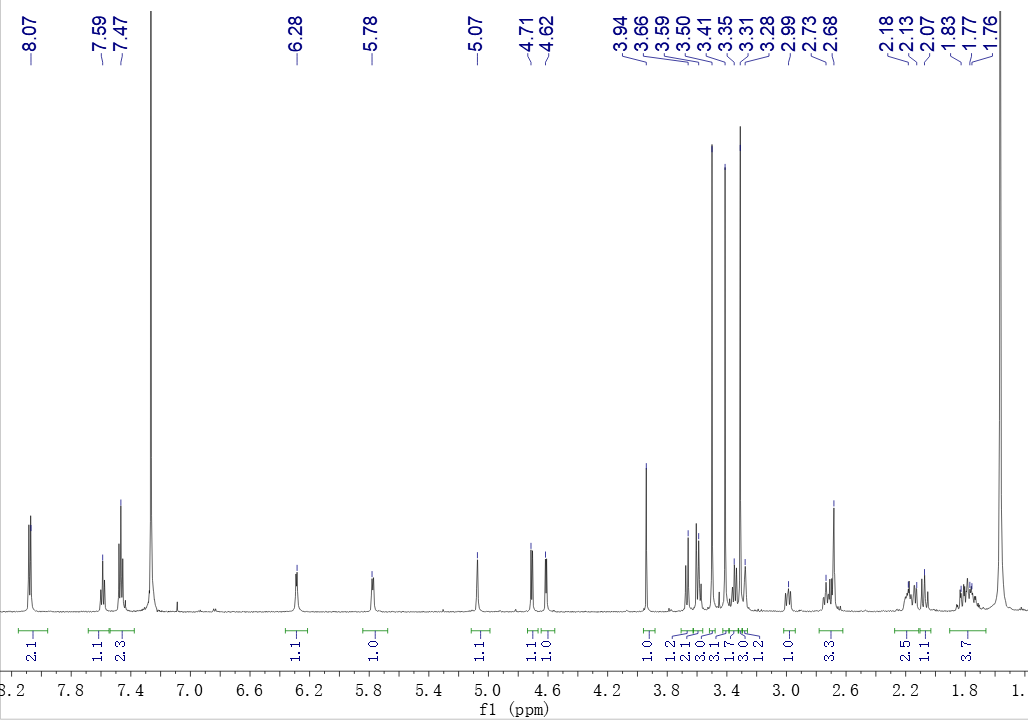


**Fig. S1** ^1^H NMR spectrum of (**1**) in CDCl_3_

**Fig. S2** ^13^C NMR spectrum of (**1**) in CDCl_3_


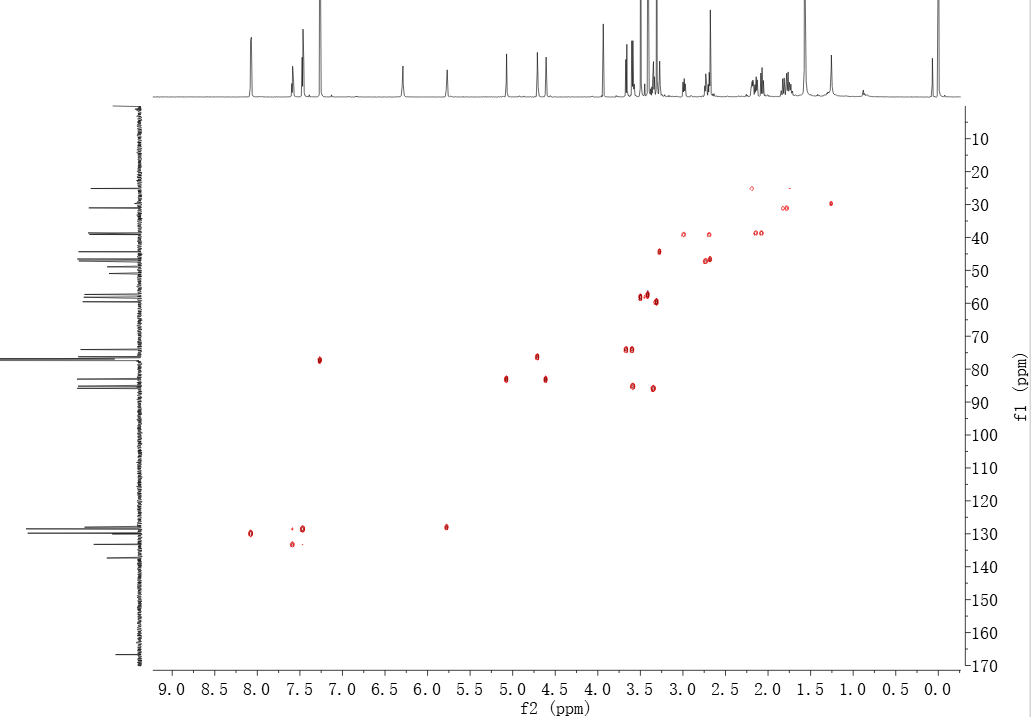


**Fig. S3** HSQC spectrum of (**1**) in CDCl_3_


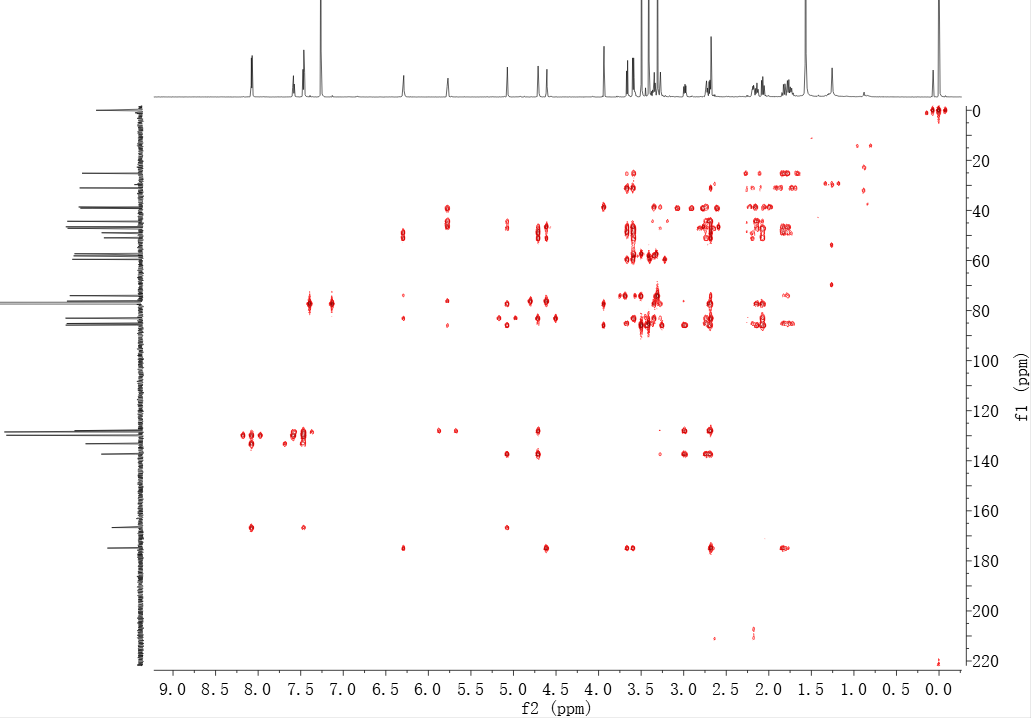


**Fig. S4** HMBC spectrum of (**1**) in CDCl_3_


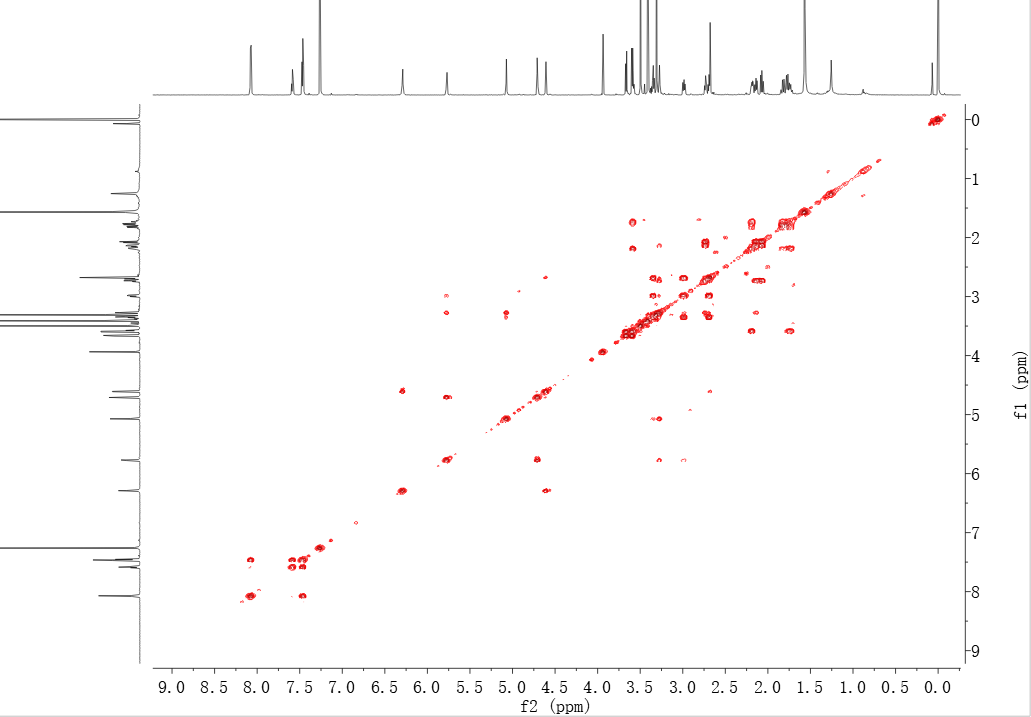


**Fig. S5** ^1^H-^1^H COSY spectrum of (**1**) in CDCl_3_


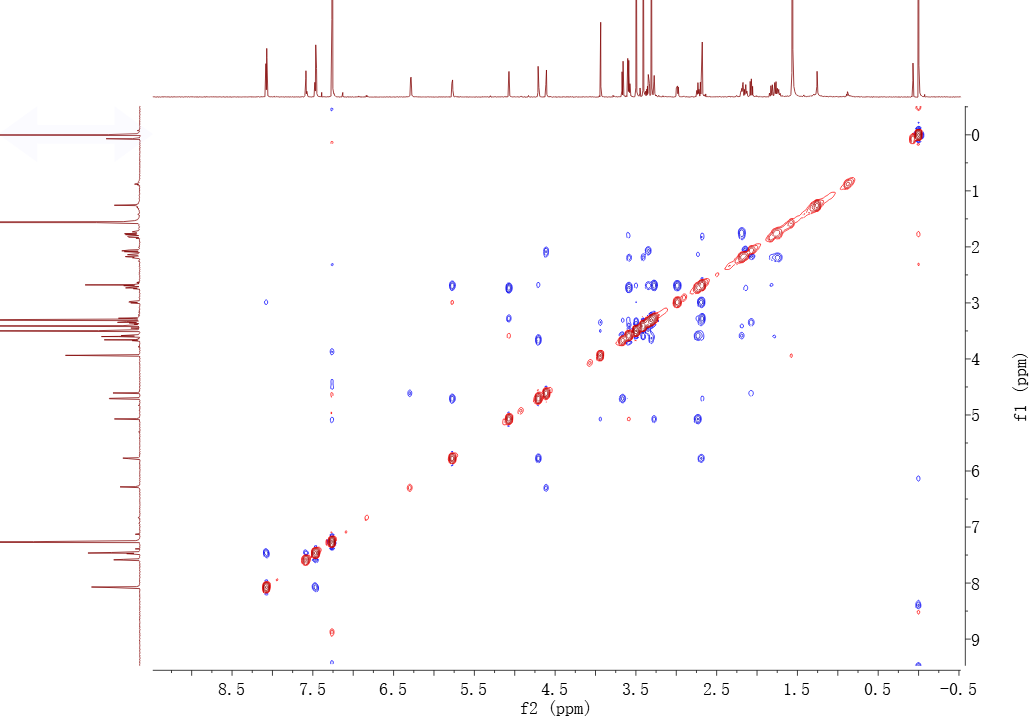


**Fig. S6** ROESY spectrum of (**1**) in CDCl_3_


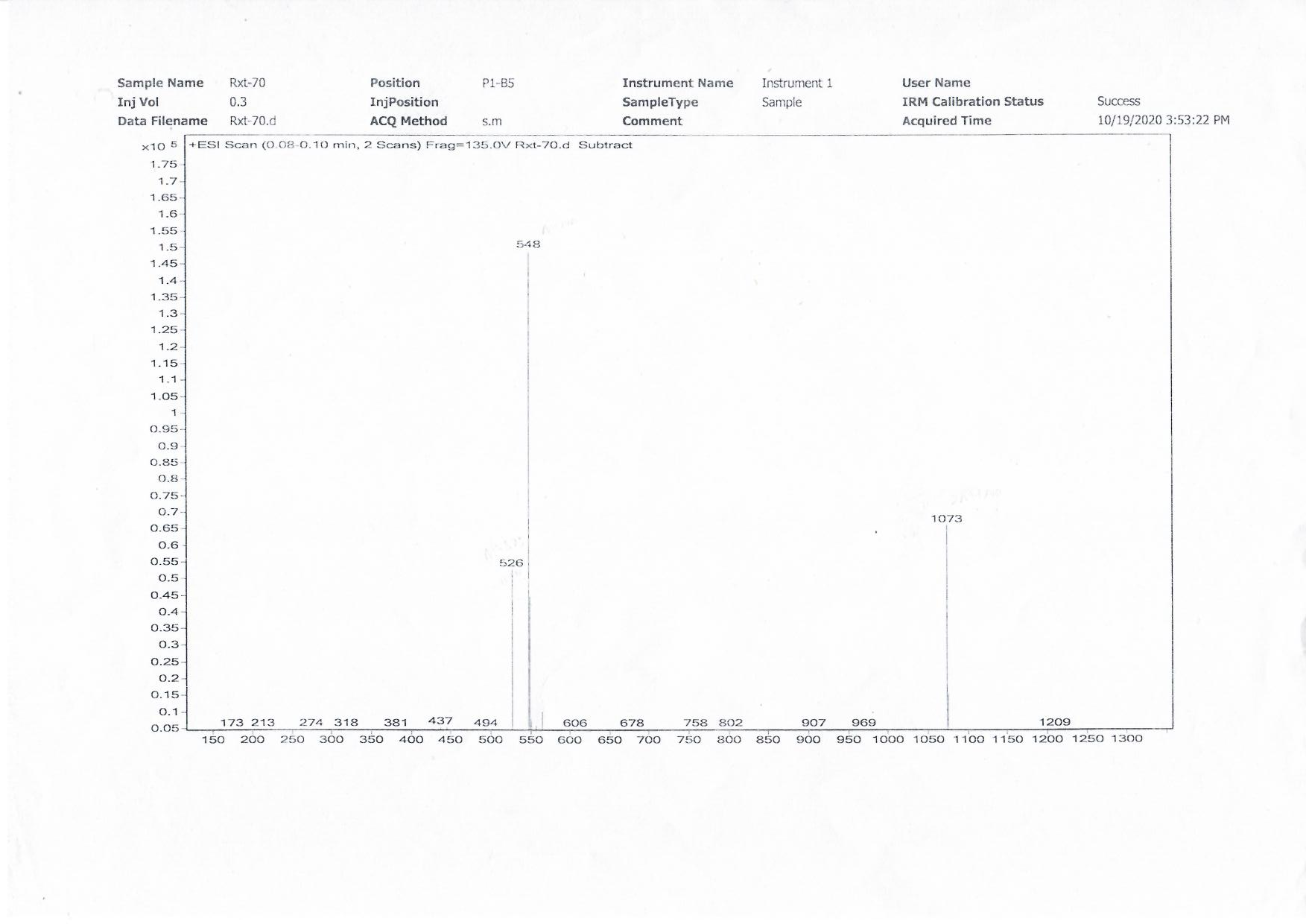


**Fig. S7** ESIMS spectroscopic report of (**1**)


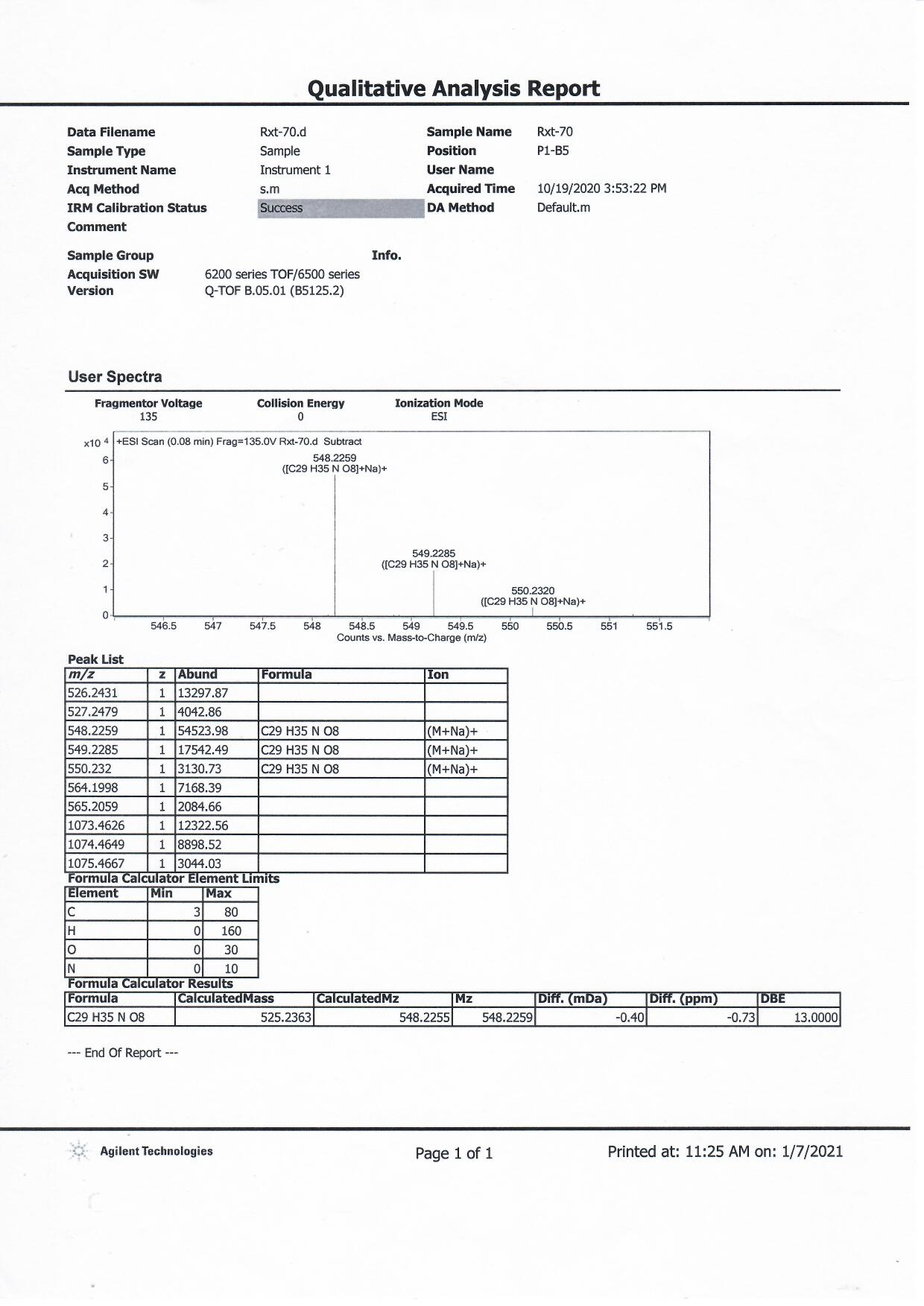


**Fig. S8** HRESIMS spectroscopic report of (**1**)


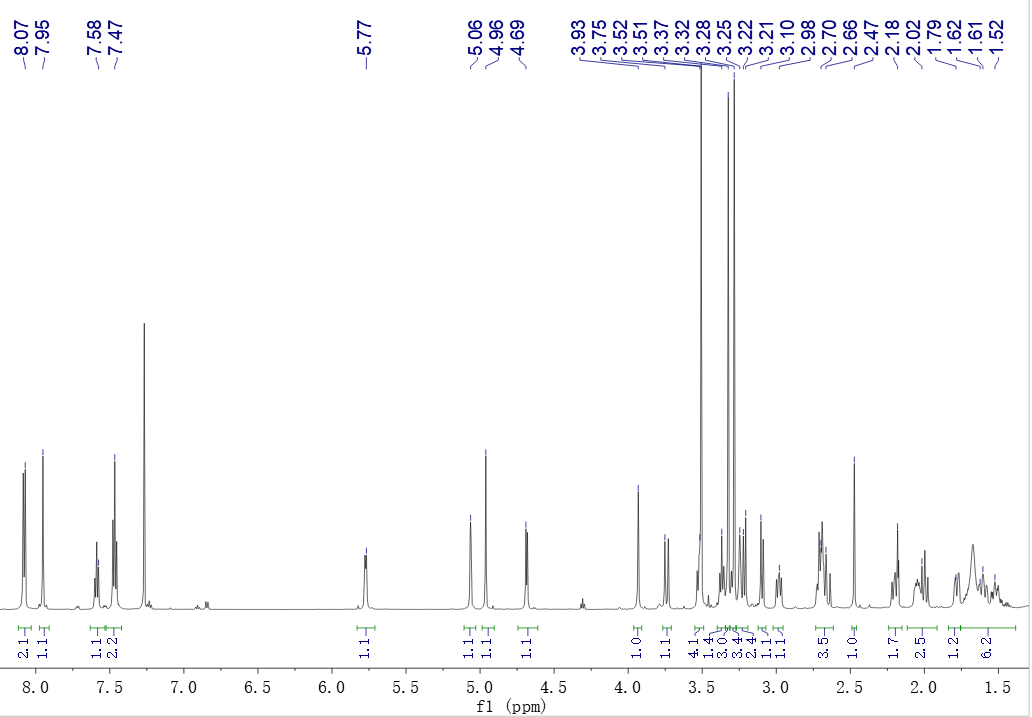


**Fig. S9** ^1^H NMR spectrum of (**2**) in CDCl_3_


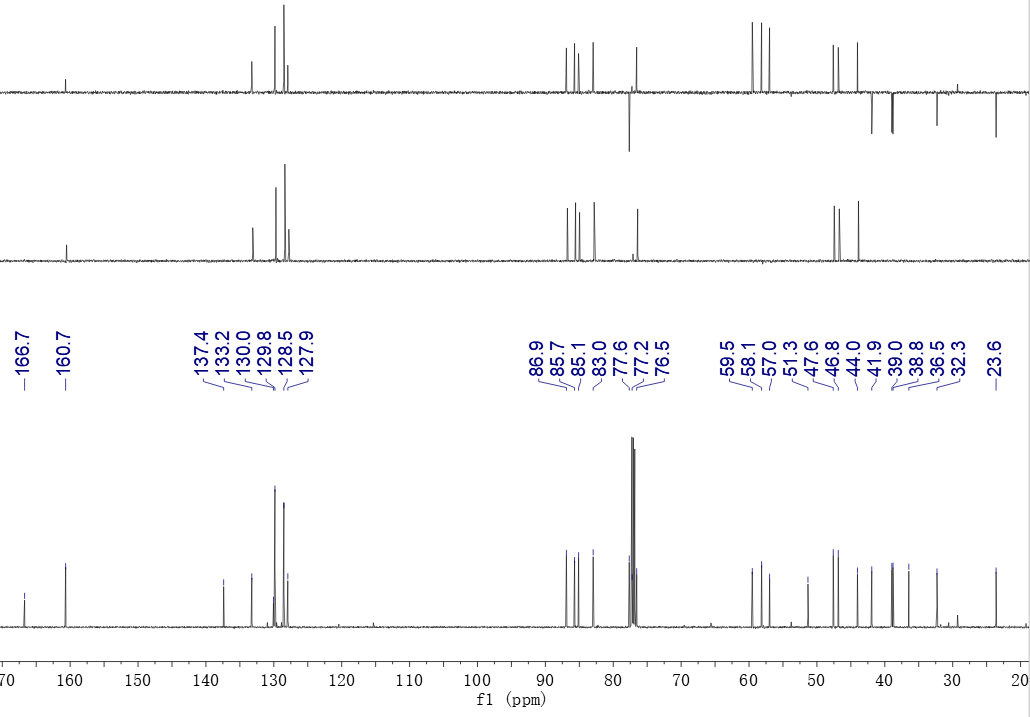


**Fig. S10** ^13^C NMR spectrum of (**2**) in CDCl_3_


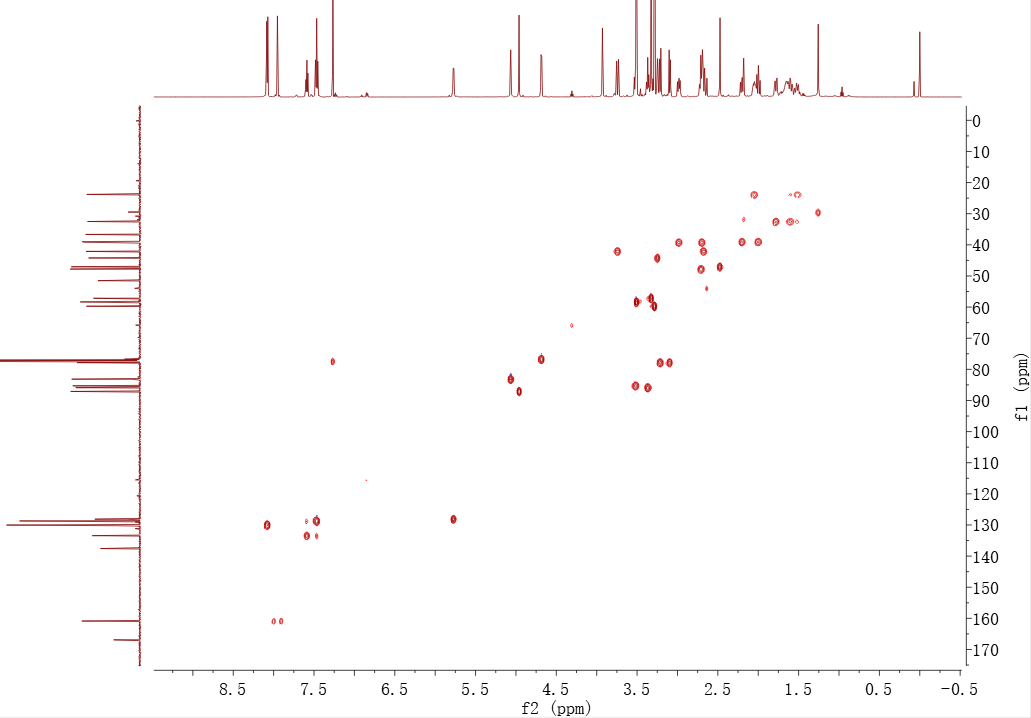


**Fig. S11** HSQC spectrum of (**2**) in CDCl_3_


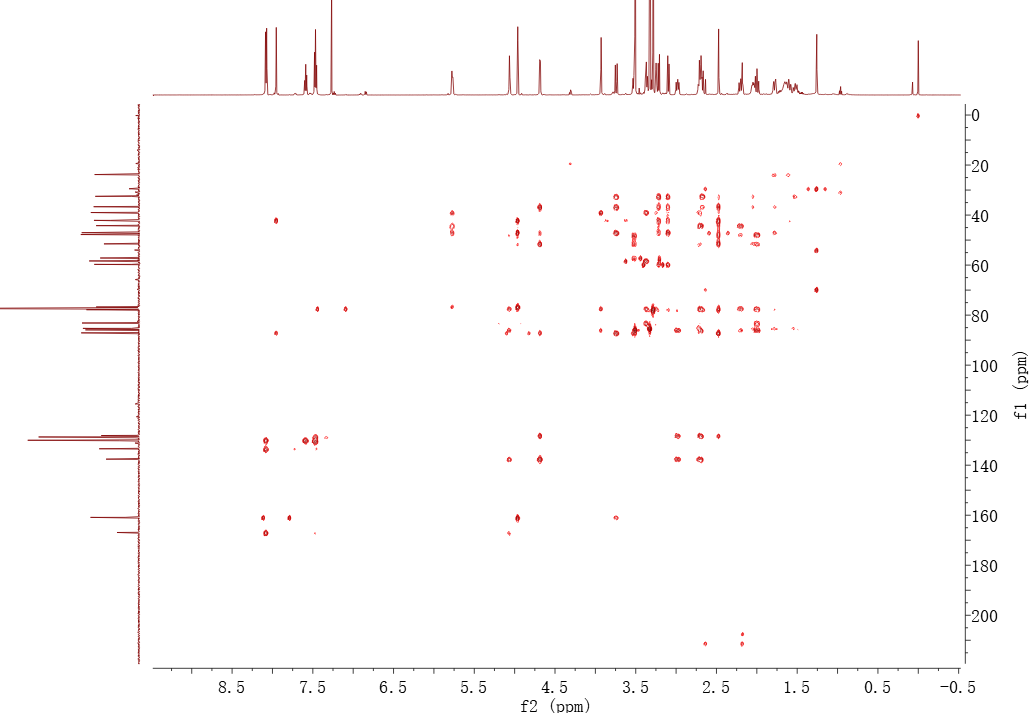


**Fig. S12** HMBC spectrum of (**2**) in CDCl_3_


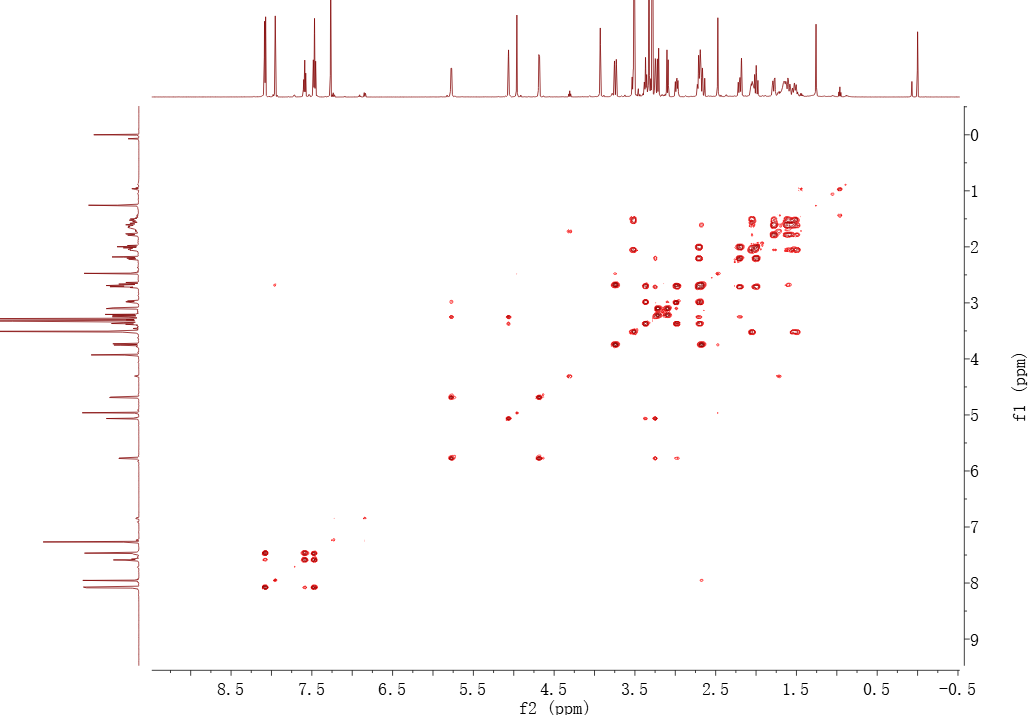


**Fig. S13** ^1^H-^1^H COSY spectrum of (**2**) in CDCl_3_


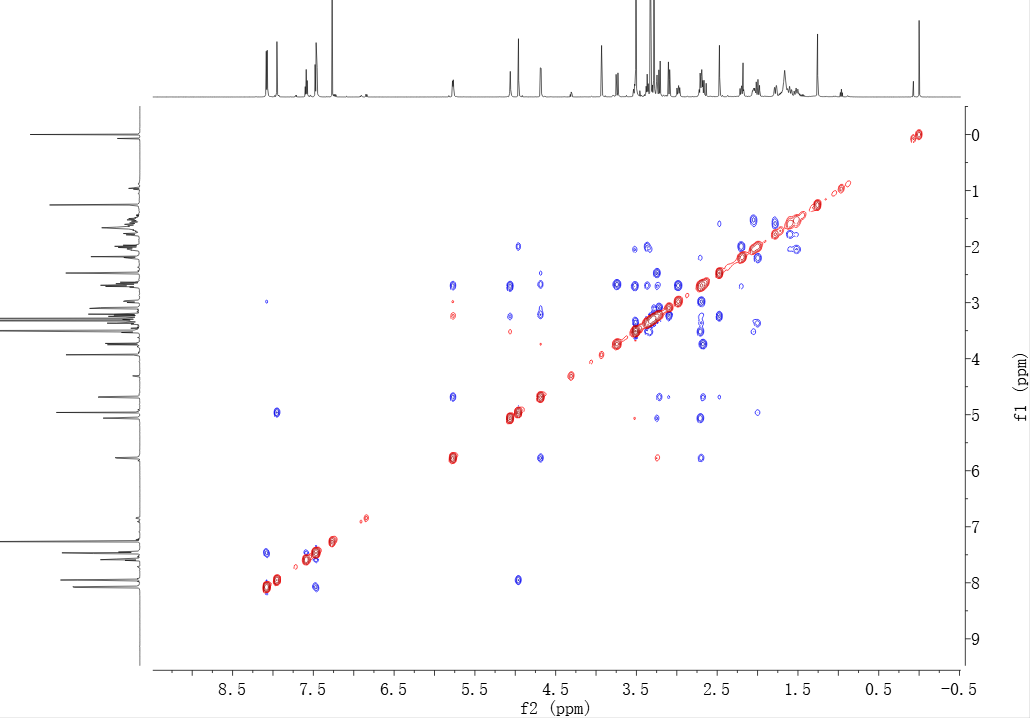


**Fig. S14** ROESY spectrum of (**2**) in CDCl_3_


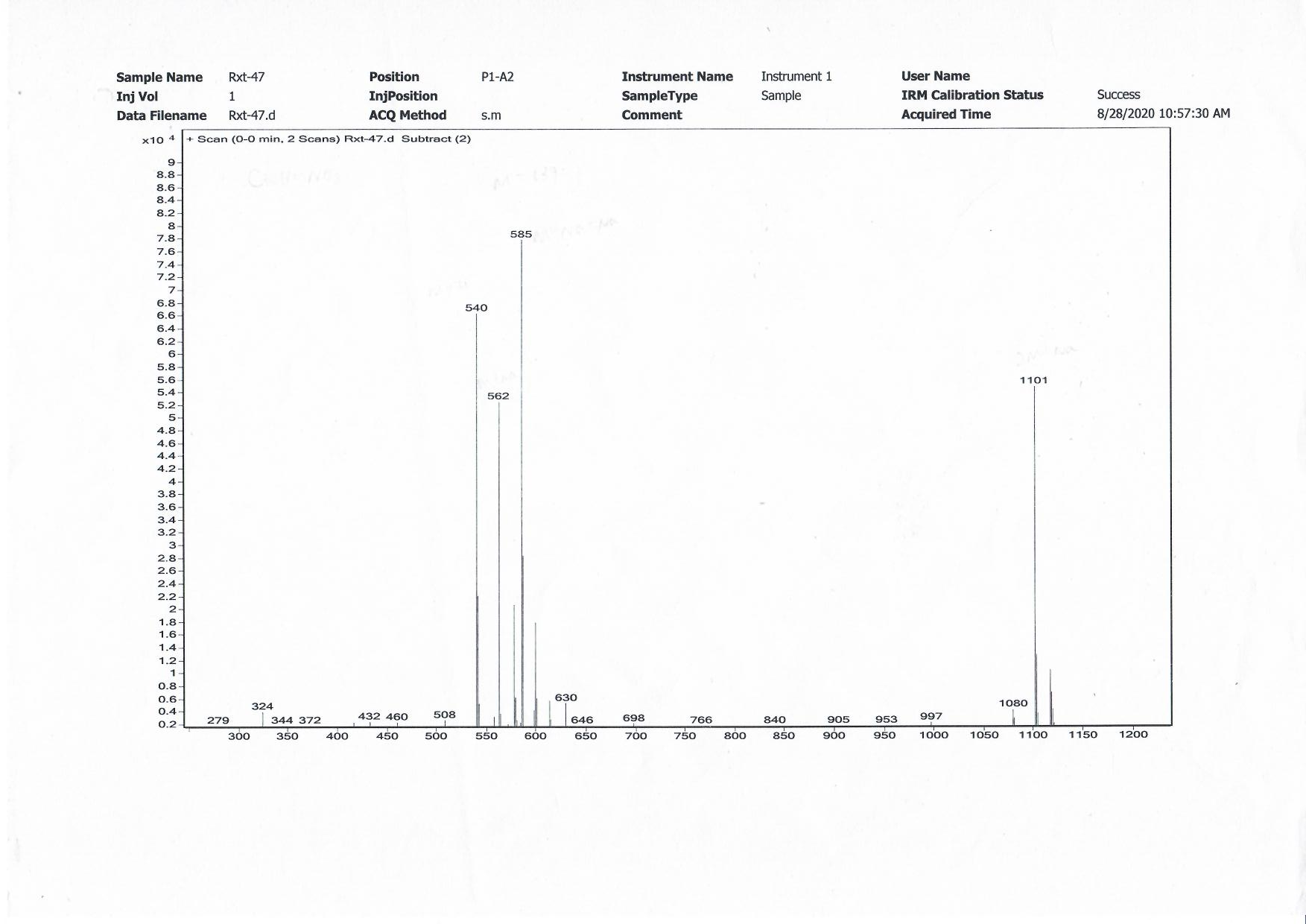


**Fig. S15** ESIMS spectroscopic report of (**2**)


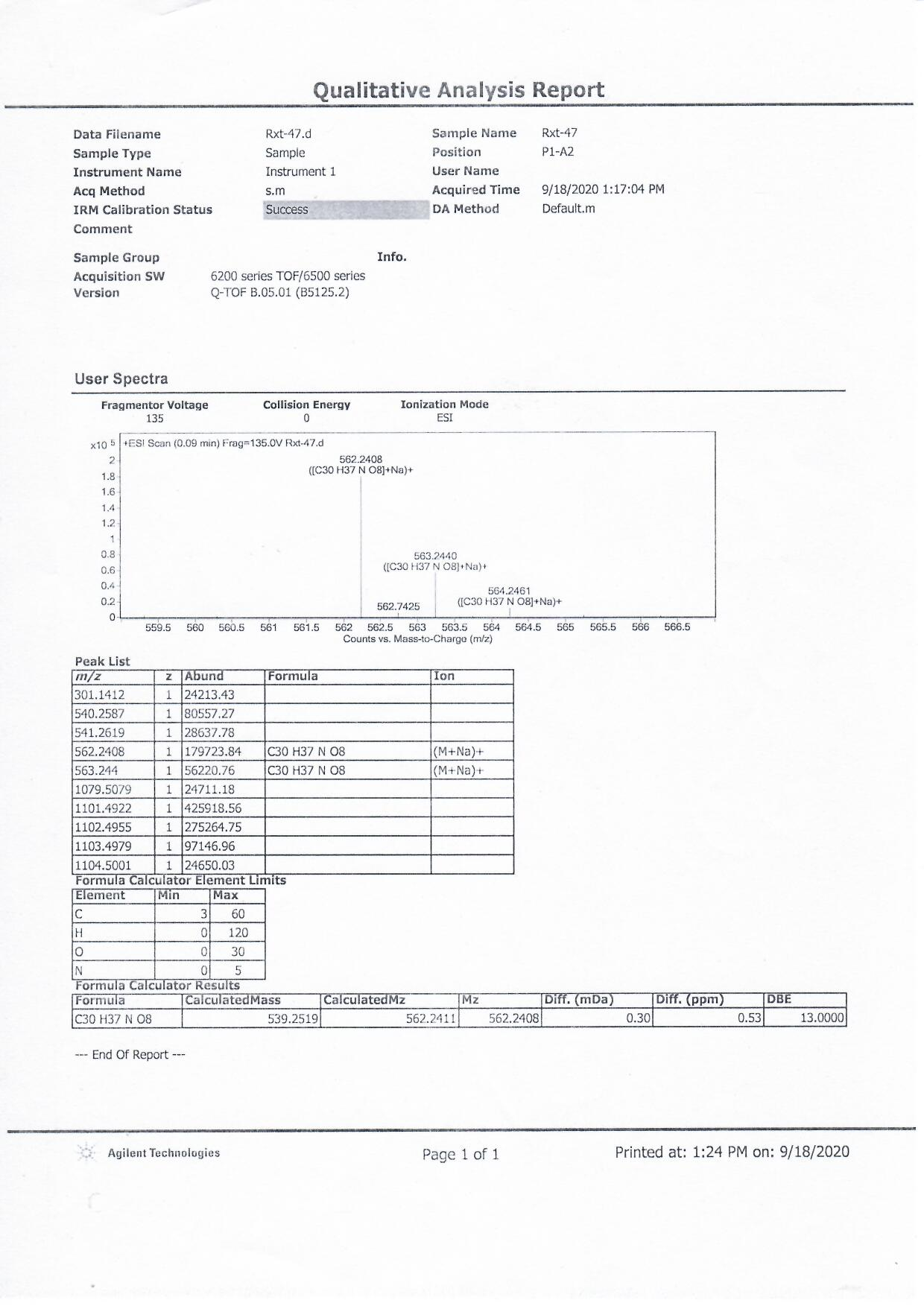


**Fig. S16** HRESIMS spectroscopic report of (**2**)


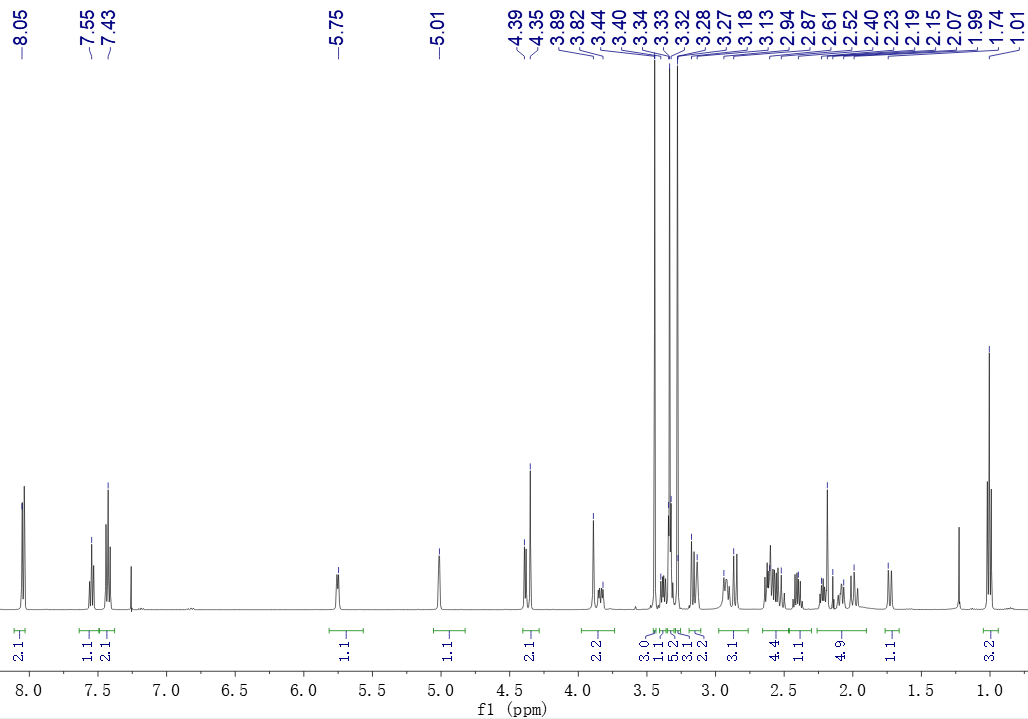


**Fig. S17** ^1^H NMR spectrum of (**3**) in CDCl_3_


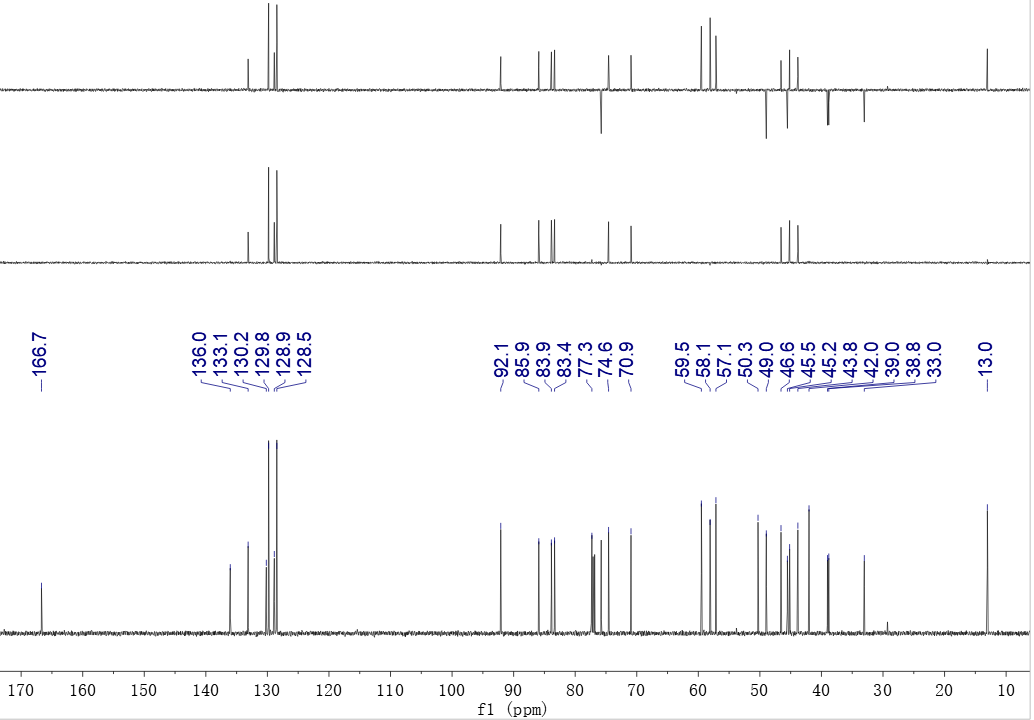


**Fig. S18** ^13^C NMR spectrum of (**3**) in CDCl_3_


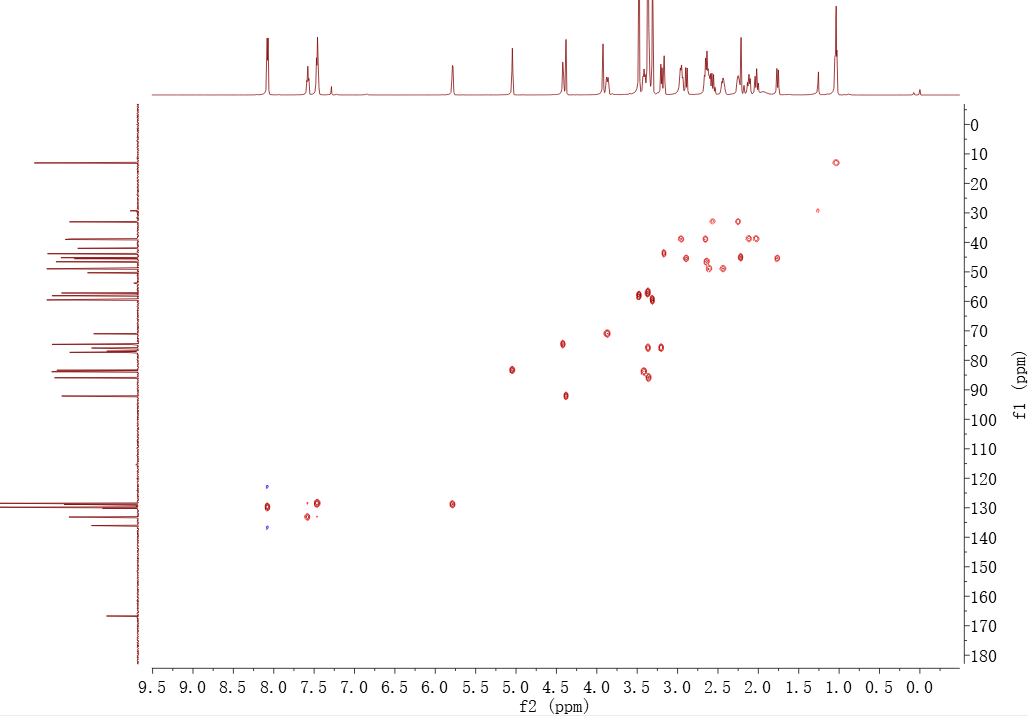


**Fig. S19** HSQC spectrum of (**3**) in CDCl_3_


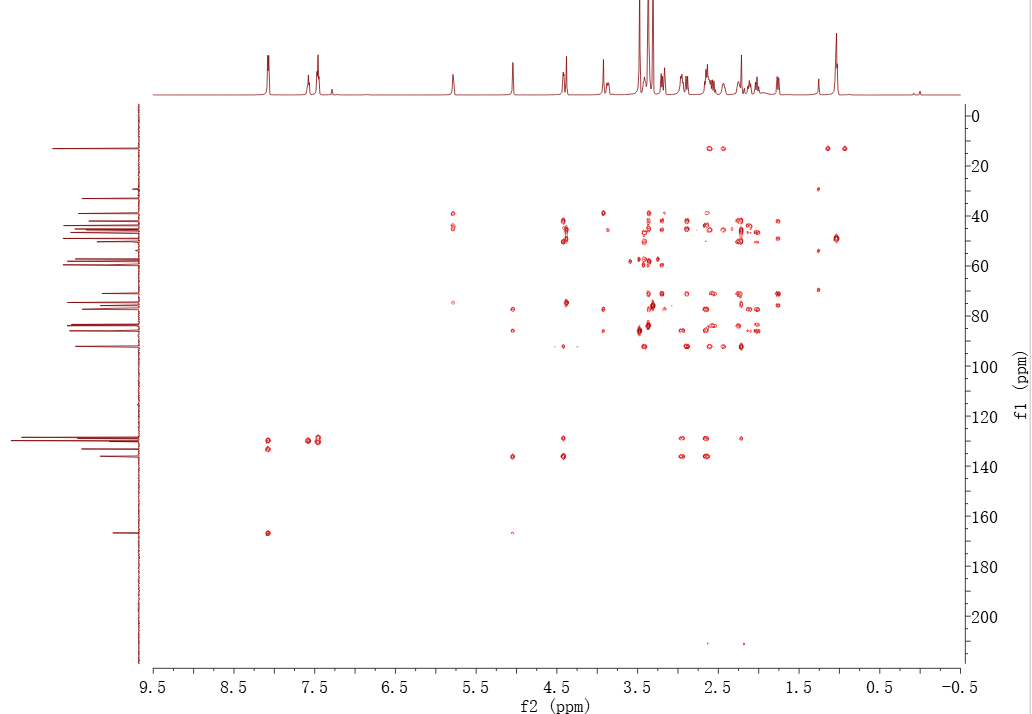


**Fig. S20** HMBC spectrum of (**3**) in CDCl_3_


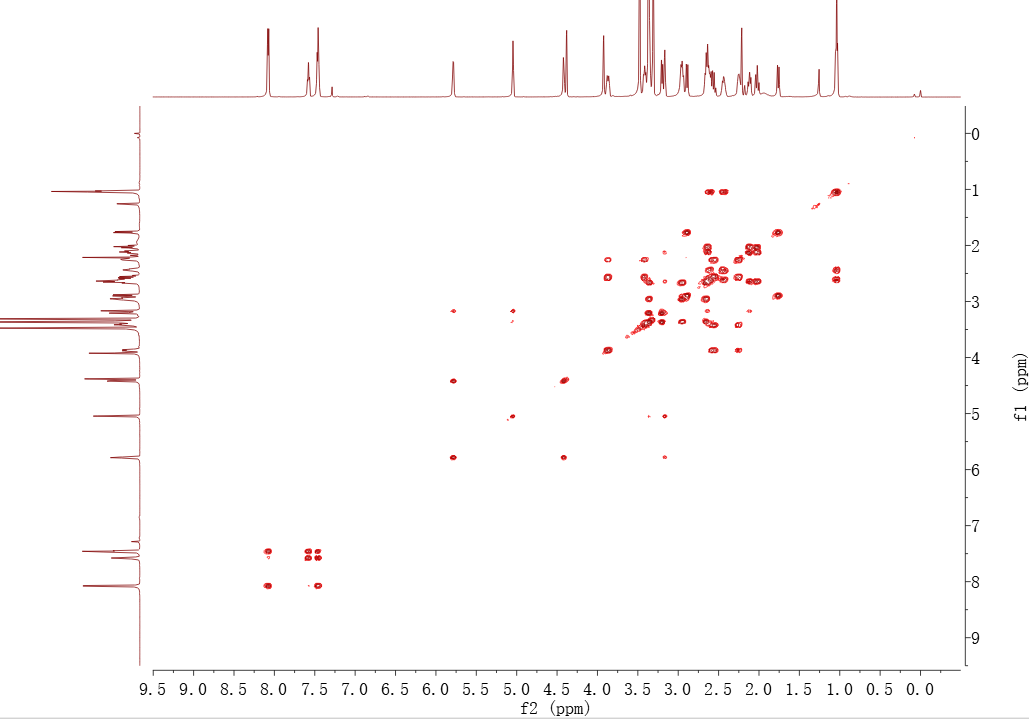


**Fig. S21** ^1^H-^1^H COSY spectrum of (**3**) in CDCl_3_


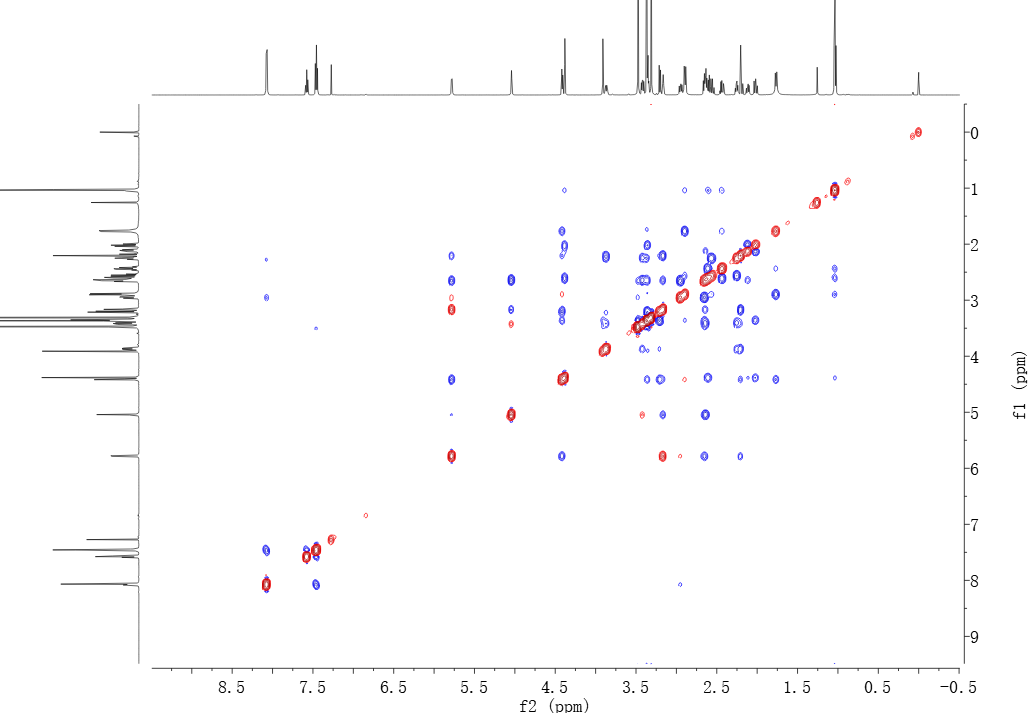


**Fig. S22** ROESY spectrum of (**3**) in CDCl_3_

#
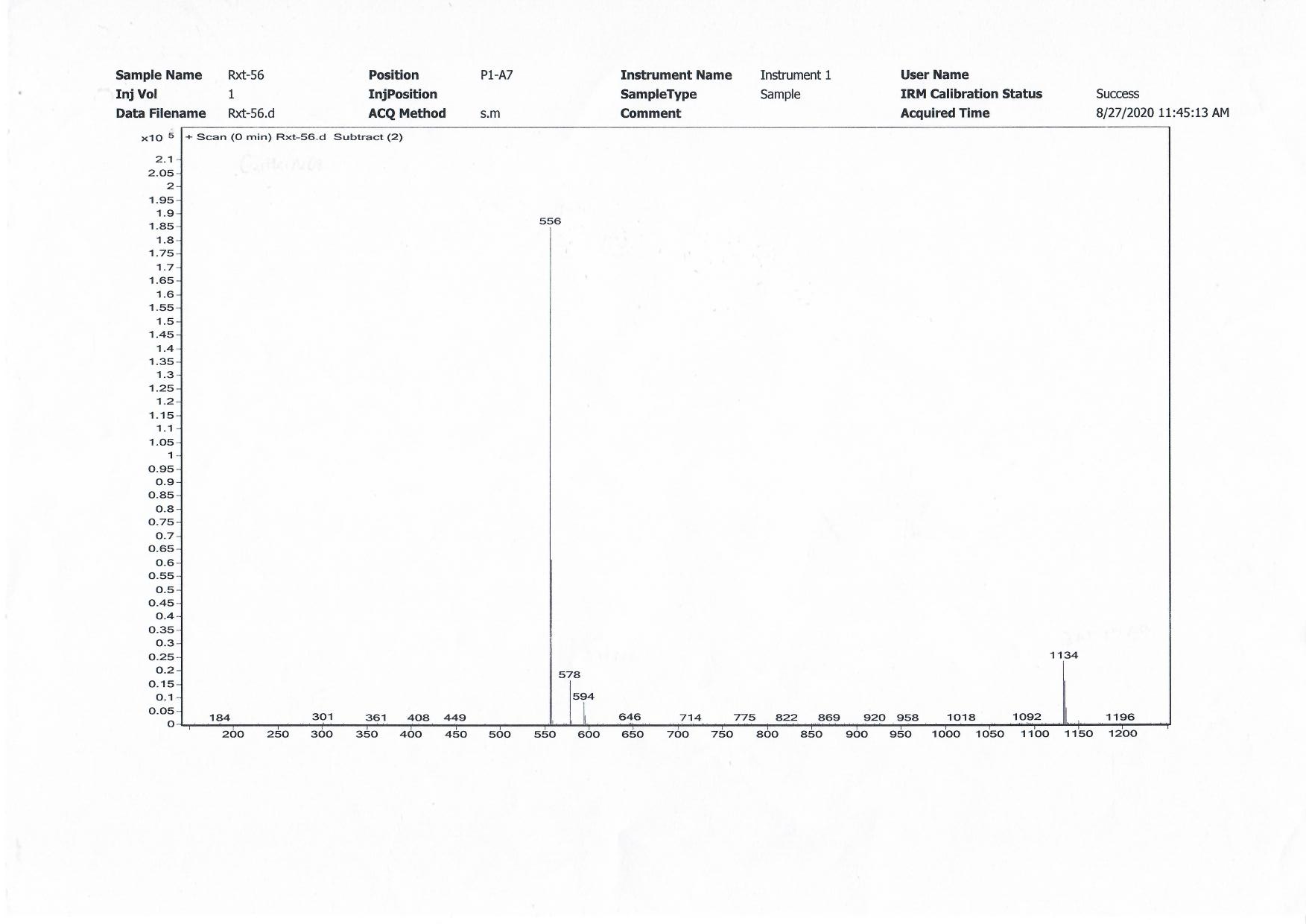
Fig. S23 ESIMS spectroscopic report of (3)


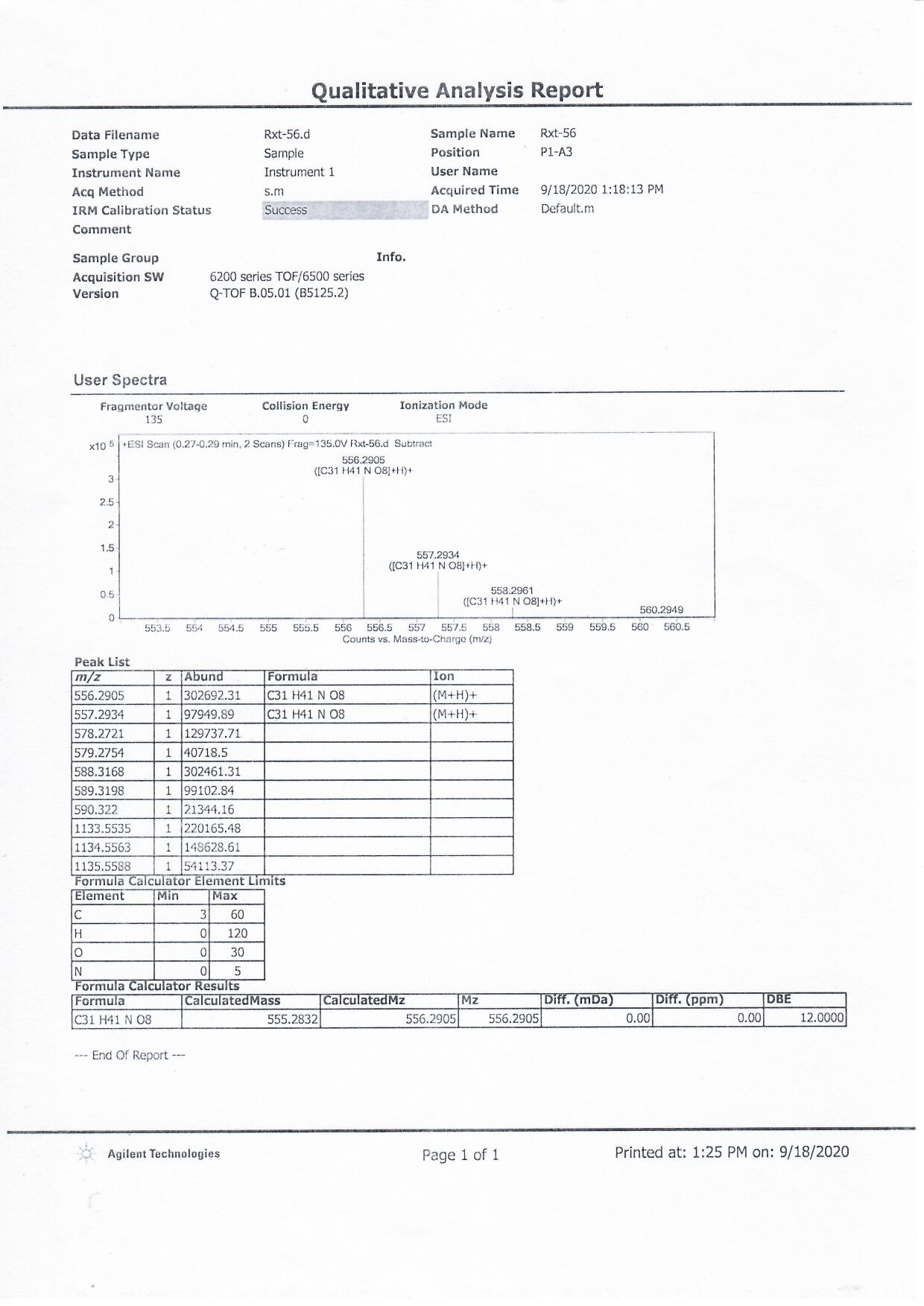


**Fig. S24** HRESIMS spectroscopic report of (**3**)

**
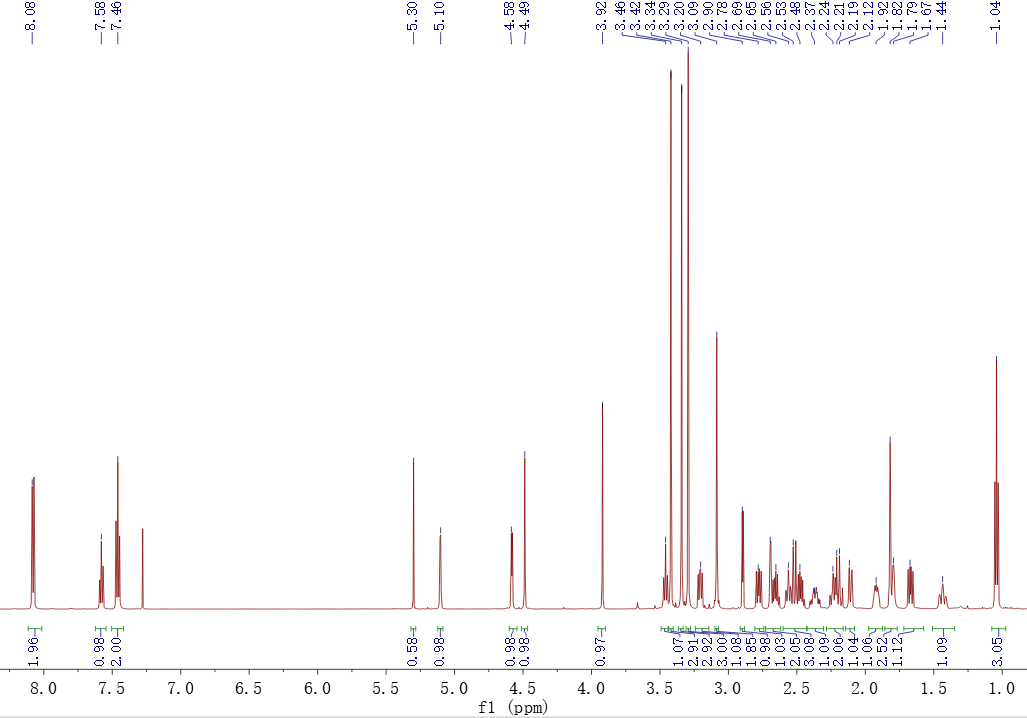
**

**Fig. S25** ^1^H NMR spectrum of (**4**) in CDCl_3_


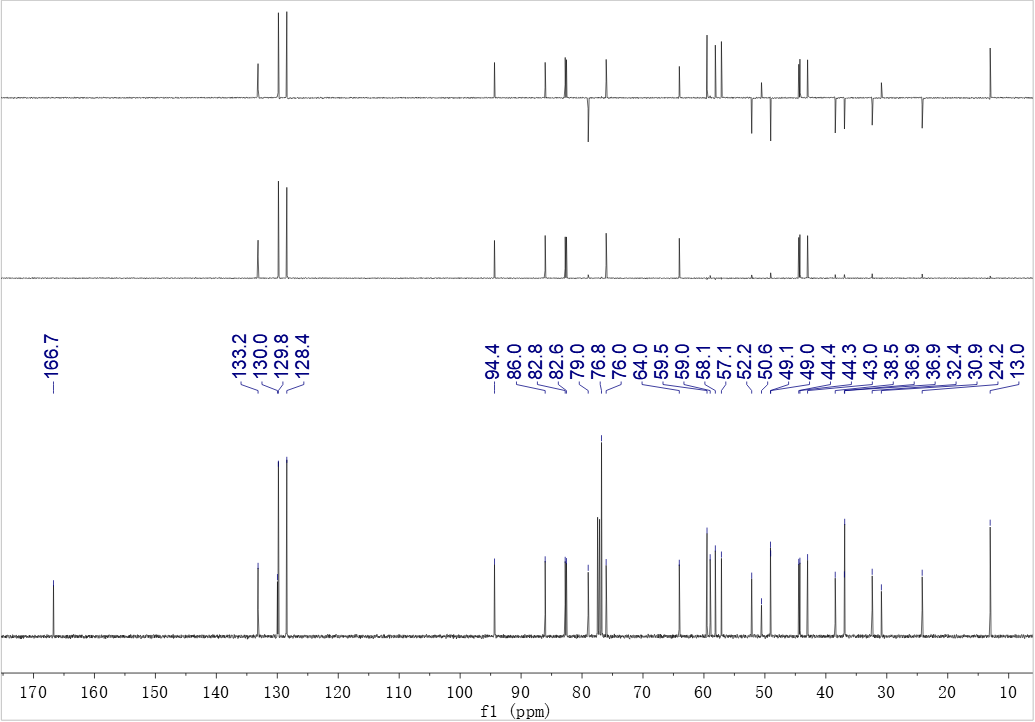


**Fig. S26** ^13^C NMR spectrum of (**4**) in CDCl_3_

**
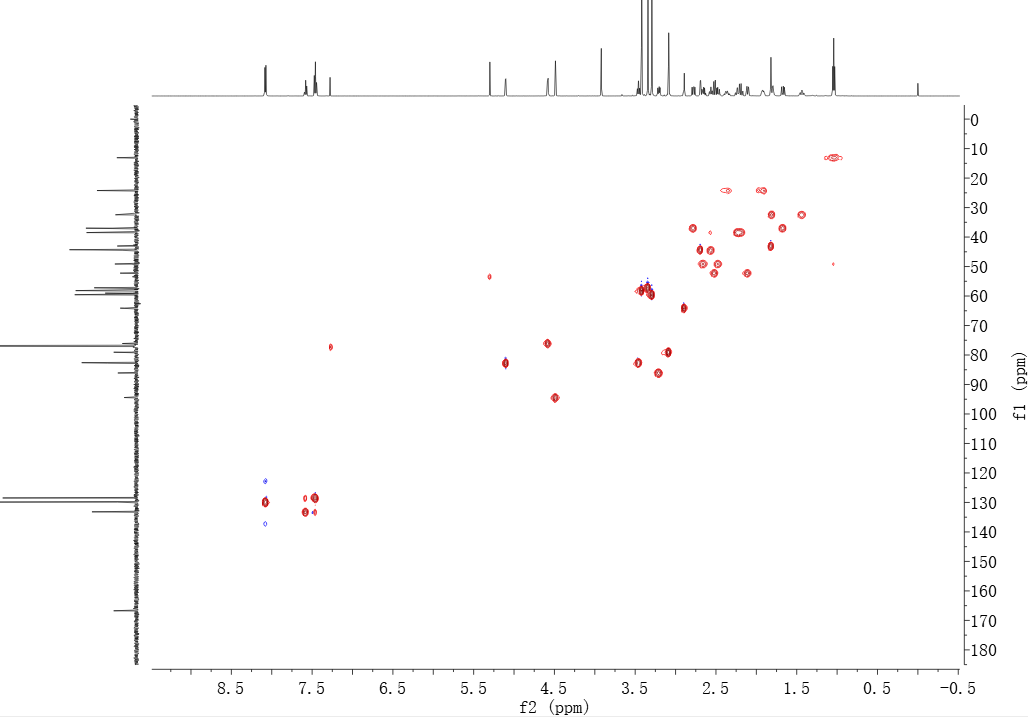
**

**Fig. S27** HSQC spectrum of (**4**) in CDCl_3_


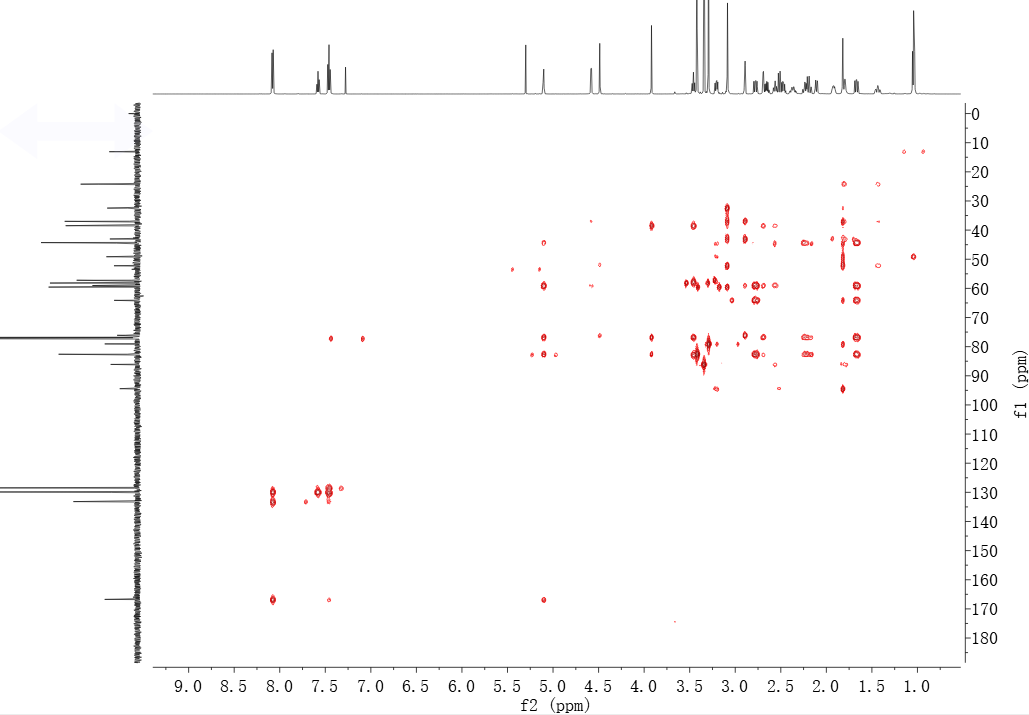


**Fig. S28** HMBC spectrum of (**4**) in CDCl_3_

**
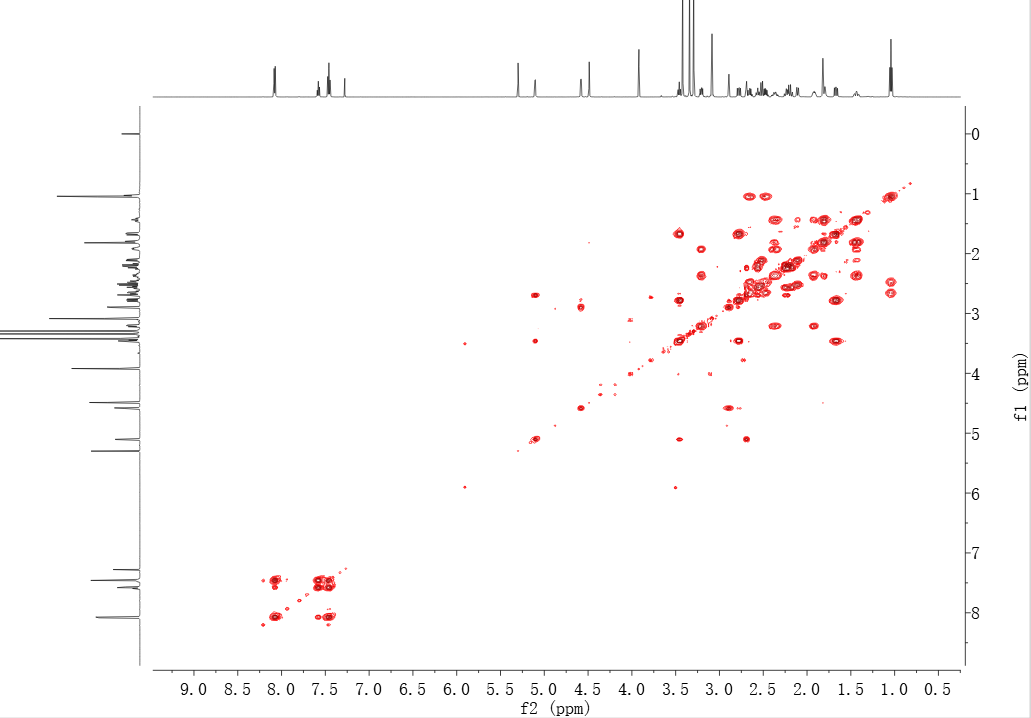
Fig. S29** ^1^H-^1^H COSY spectrum of (**4**) in CDCl_3_


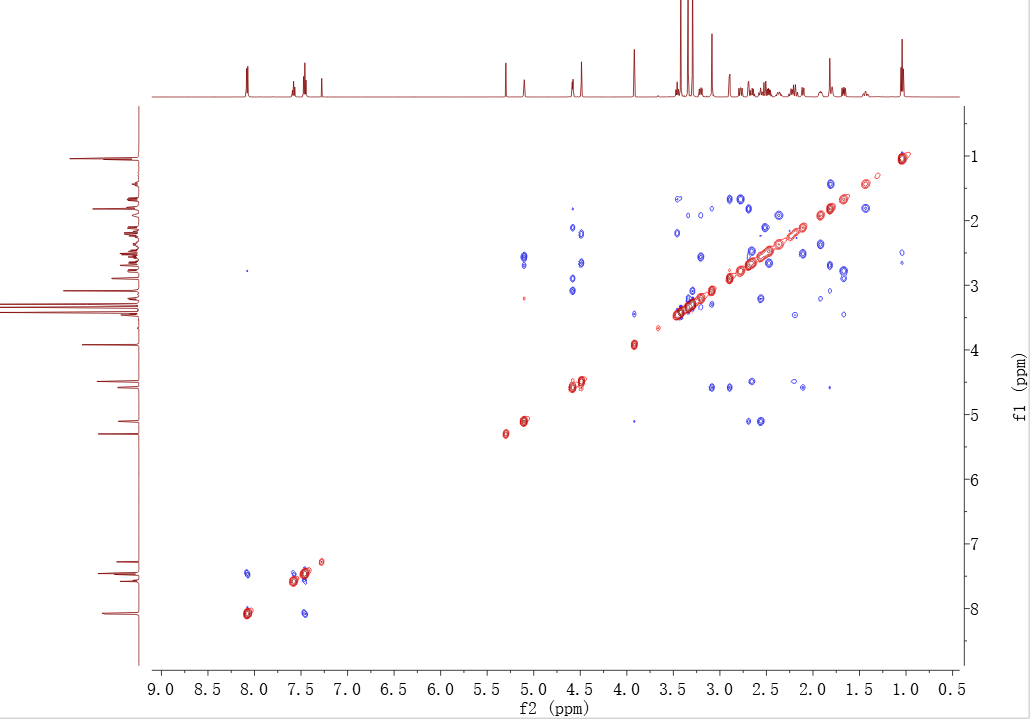


**Fig. S30** ROESY spectrum of (**4**) in CDCl_3_


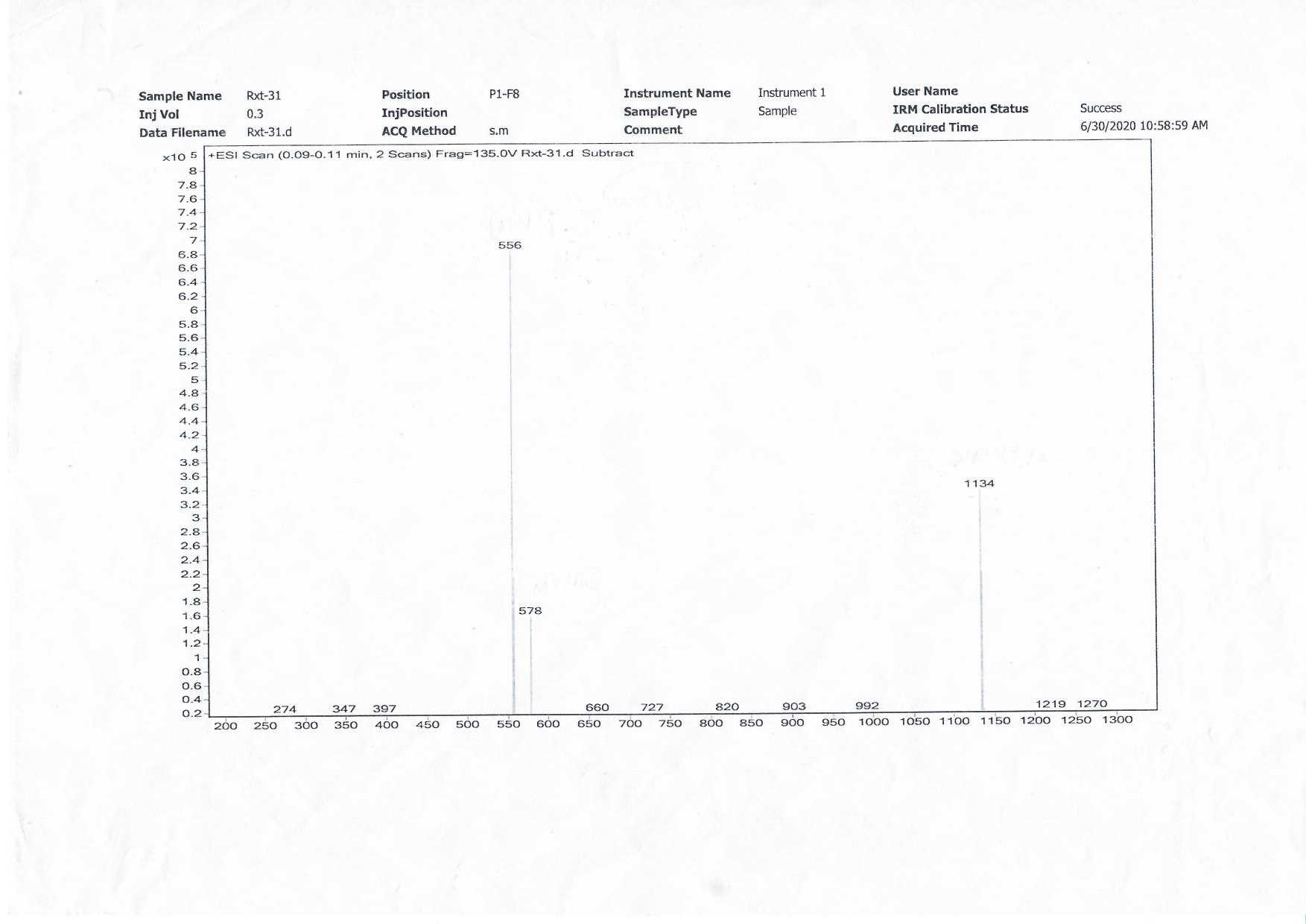


**Fig. S31** ESIMS spectroscopic report of (**4**)


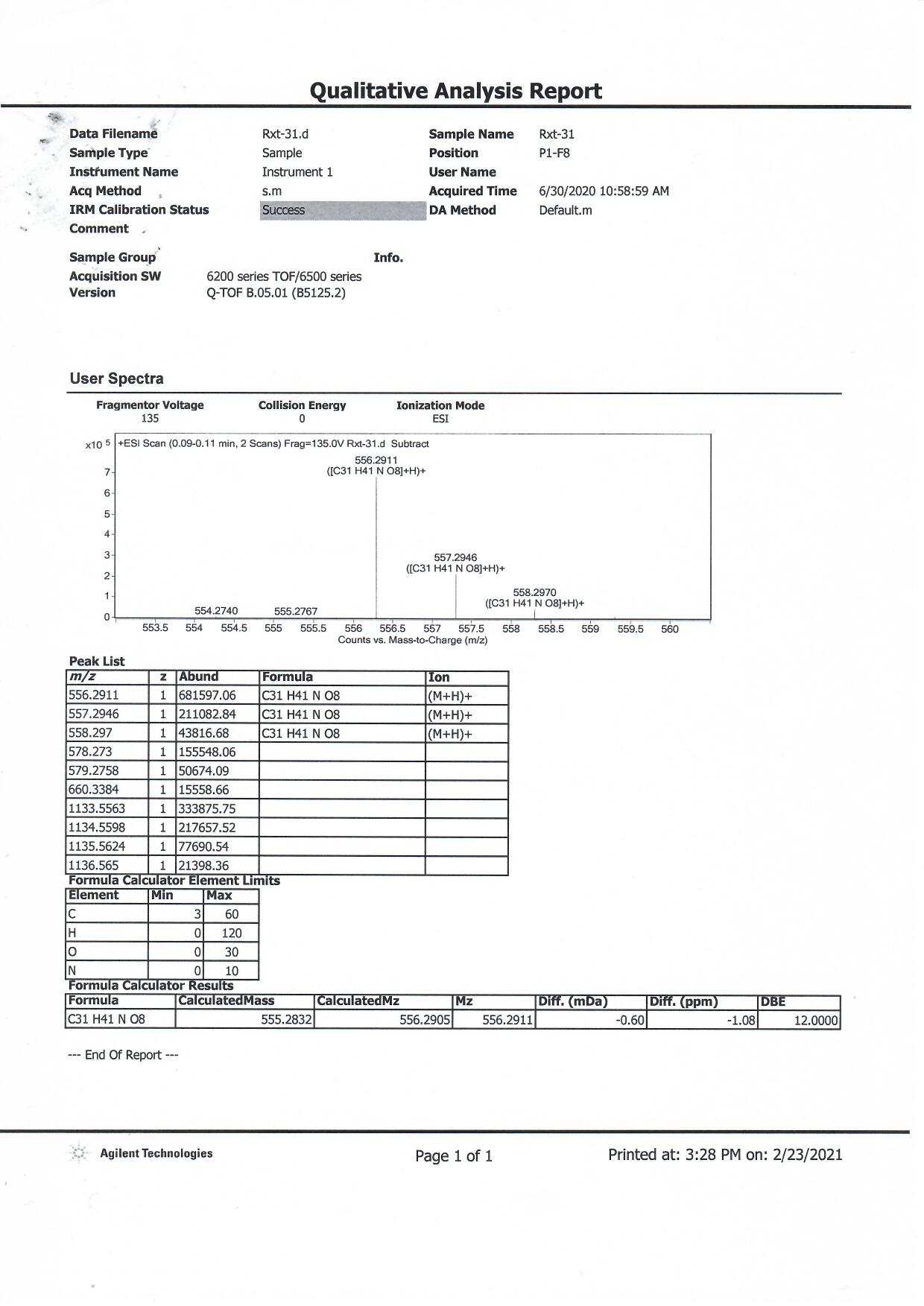


**Fig. S32** HRESIMS spectroscopic report of (**4**)


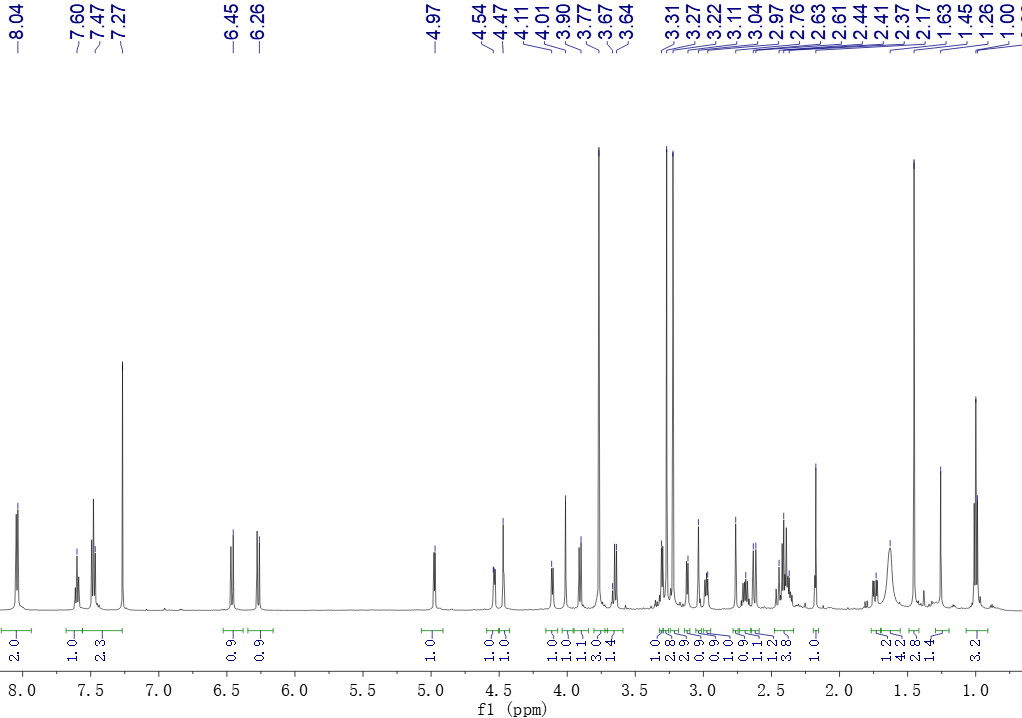


**Fig. S33** ^1^H NMR spectrum of (**5**) in CDCl_3_


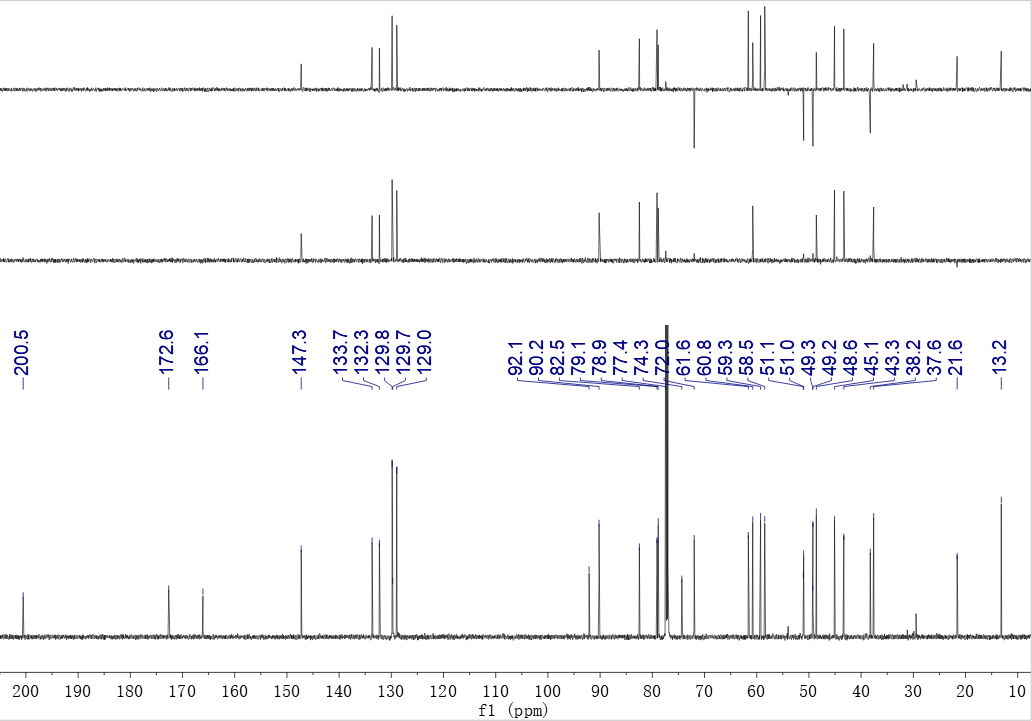


**Fig. S34** ^13^C NMR spectrum of (**5**) in CDCl_3_


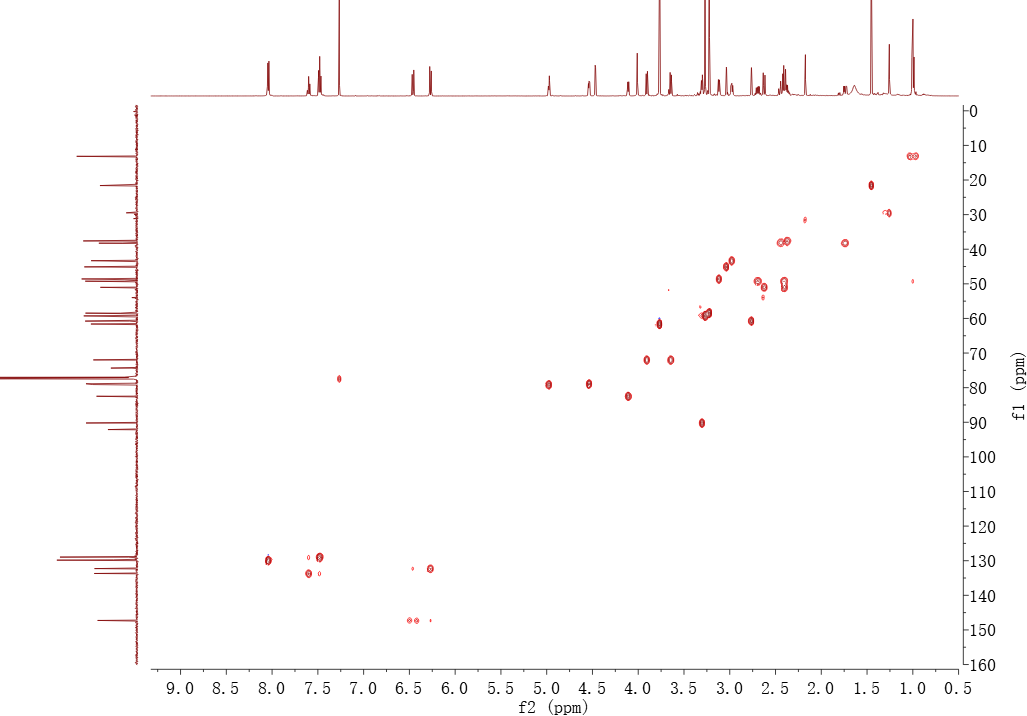


**Fig. S35** HSQC spectrum of (**5**) in CDCl_3_


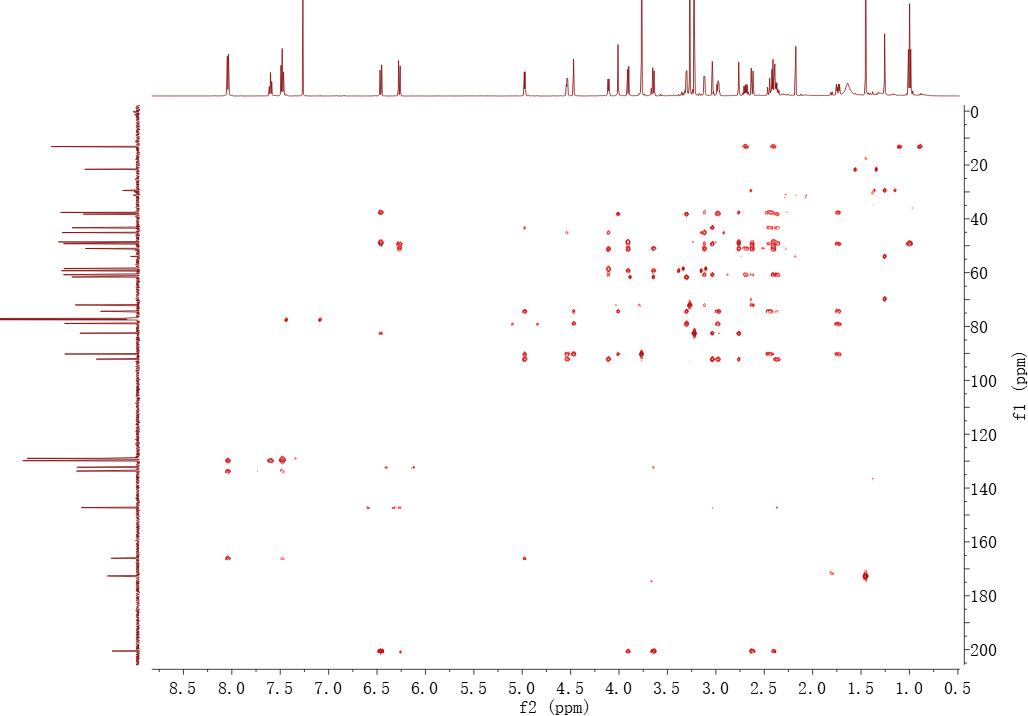


**Fig. S36** HMBC spectrum of (**5**) in CDCl_3_


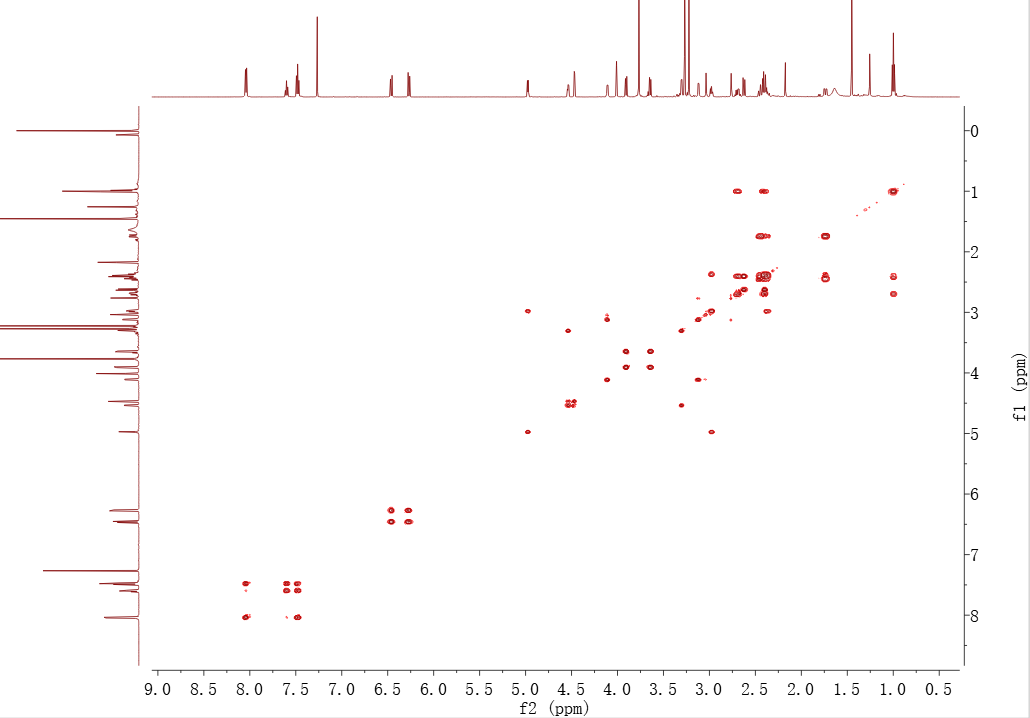


**Fig. S37** ^1^H-^1^H COSY spectrum of (**5**) in CDCl_3_


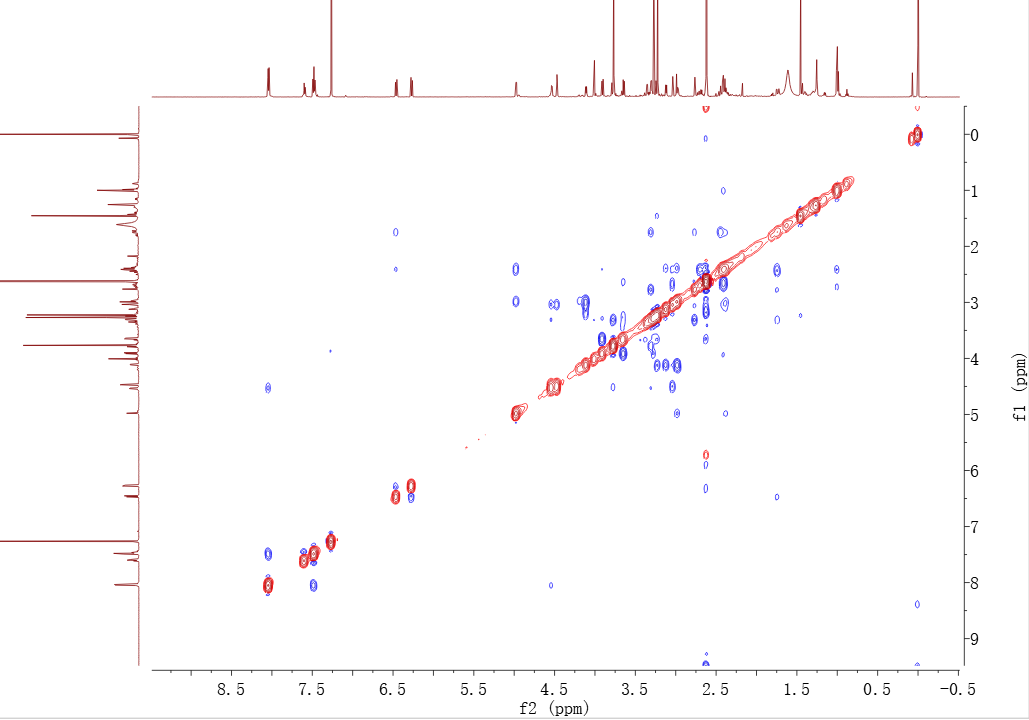


**Fig. S38** ROESY spectrum of (**5**) in CDCl_3_


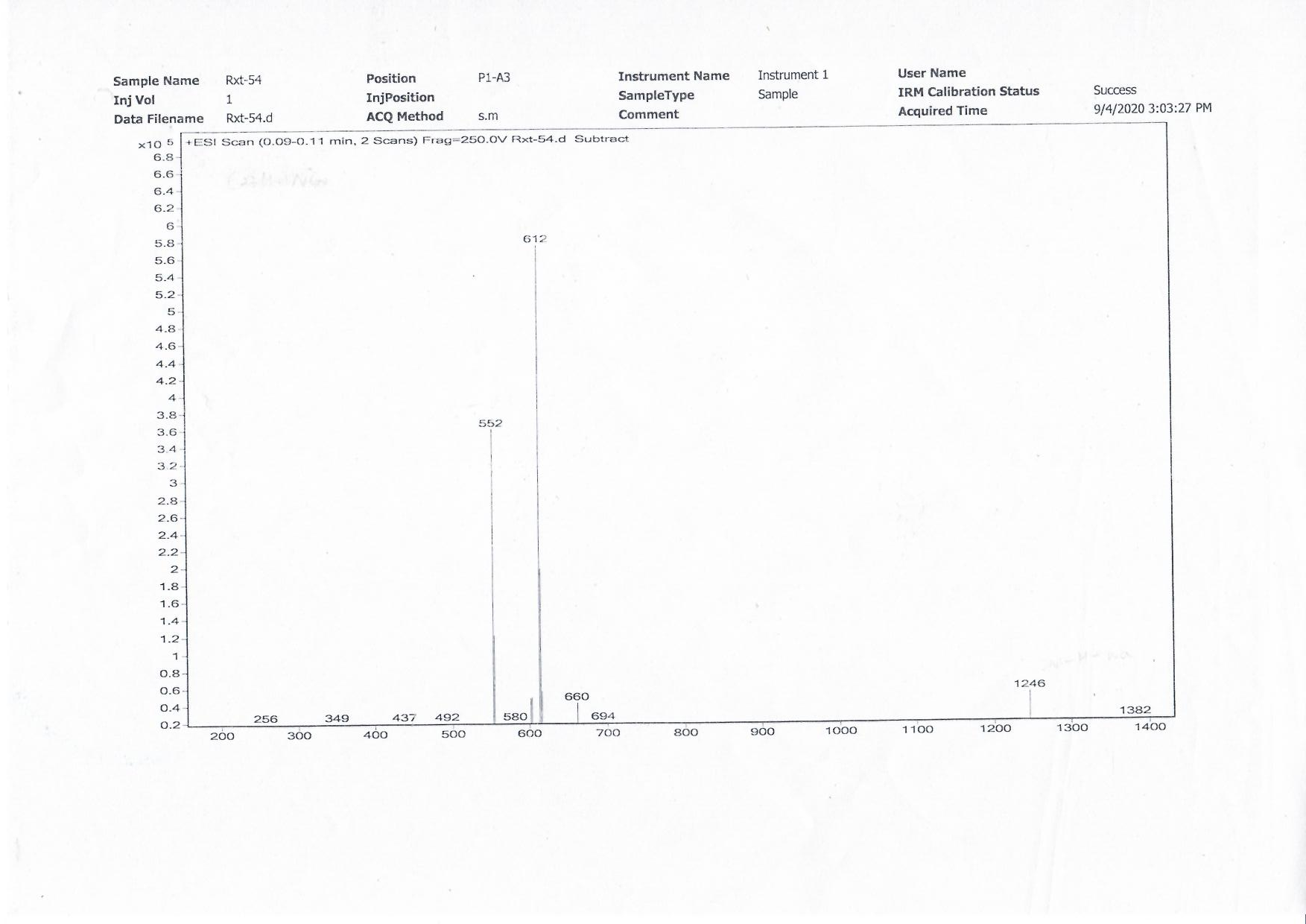


**Fig. S39** ESIMS spectroscopic report of (**5**)

**Fig. S39** ESIMS spectroscopic report of (**5**)


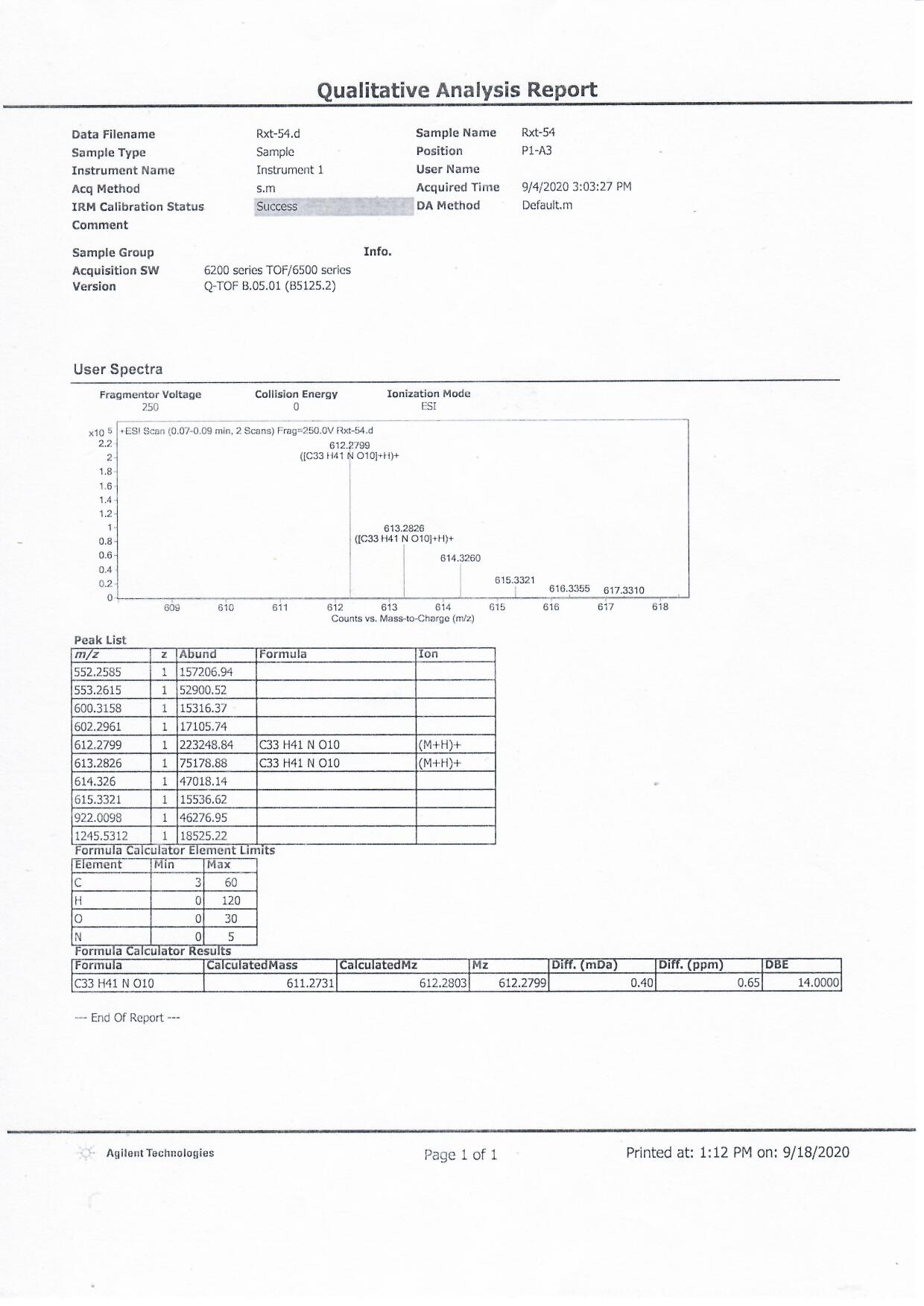


**Fig. S40** HRESIMS spectroscopic report of (**5**)
